# Integrative transcriptomic and metabolomic analysis reveals key genes and regulatory networks underlying seed germination in *Polygonatum sibiricum*

**DOI:** 10.1186/s12864-026-12803-x

**Published:** 2026-04-15

**Authors:** Wenchang Gou, Xinfei Zhang, Xiaoqi Zhang, Qian Wang, Zejing Yang, Ji Li, Jianguo Meng, Junfeng Niu, Zhezhi Wang, Shiqiang Wang

**Affiliations:** 1 National Engineering Laboratory for Resource Development of Endangered Crude Drugs in Northwest China, Shaanxi Normal University, Xi’an, 710119 China; 2 Key Laboratory of Medicinal Resources and Natural Pharmaceutical Chemistry, Shaanxi Normal University, The Ministry of Education, Xi’an, 710119 China; 3 College of Life Sciences, Shaanxi Normal University, Xi’an, 710119 China; 4 Shaanxi Panlong Pharmaceutical Group Limited By Share Ltd, Xi’an, China; 5 Hinggan League Institute of Agricultural and Husbandry Sciences, Ulanhot, China

**Keywords:** *Polygonatum sibiricum* Red, Seed germination, Gene expression, Metabolites

## Abstract

**Background:**

*Polygonatum sibiricum* Red. (*P. sibiricum*) seeds exhibit strong dormancy, which prolongs germination period, reduces germination rate, and thus hinders its production. Research on breaking seed dormancy via optimized sand storage and clarifying germination-related mechanisms is insufficient. This study focused on optimizing sand storage conditions of *P. sibiricum* seeds and analyzing their biological traits, enzyme gene expressions, and metabolic changes to elucidate germination kinetics.

**Results:**

The predicted optimal conditions (GA_3_ 130.497 mg/L, 4.90 °C, 1:1 sand: Huangjiang slag) gave a theoretical germination rate of 89.65% ± 0.31%; validation experiments achieved 89.31% ± 0.34%, confirming robustness. High abscisic acid (ABA) content was detected during dormancy; GA_3_, jasmonic acid (JA) increased during seed coat penetration, with indole-3-acetic acid (IAA) rising simultaneously. RNA sequencing (RNA-seq) identified 1,710 Differentially expressed genes (DEGs) involved in cell wall modification, endosperm degradation, phytohormone synthesis/signaling, etc. Liquid Chromatography-Mass Spectrometry (LC-MS) confirmed transcriptome results, highlighting beta-1, 2-Mannotriose, Isocitric acid, dAMP, AMP, and Methyl jasmonate as germination-related metabolites. Plant secondary metabolism, amino acid synthesis, and carbohydrate metabolism pathways were enriched during germination.

**Conclusion:**

This study determined GA_3_ concentration, temperature, and sand-slag ratio as key factors for *P. sibiricum* seed germination, providing optimal conditions with 89.31% ± 0.34% germination rate. It revealed a synergistic mechanism: ABA maintains dormancy, while GA_3_/JA/IAA promote germination; 1,710 DEGs and key metabolites regulate germination via core pathways. These findings support *P. sibiricum* seedling cultivation and industrial development.

**Supplementary Information:**

The online version contains supplementary material available at 10.1186/s12864-026-12803-x.

## Introduction

*P. sibiricum* is a perennial herb that belongs to the family Asparagaceae, which is distributed across temperate regions of the northern hemisphere, including countries such as China, Japan, Korea, India, Russia, Europe, and North America [1, 2]. Modern pharmacological studies have demonstrated that it is beneficial for the regulation of human immunity, provision of antioxidants, and reduction of blood sugar levels [3]. In China, *P. sibiricum* is renowned as a traditional medicinal herb and functional food [4, 5], as well as a health-enhancing substance [6] that is characterized by its sweet fragrance and taste. Additionally, since *P. sibiricum* has been included in the “list of items that are both food and medicine” by the National Health Commission of the People’s Republic of China, it is gradually being extensively used in health care products, as well as in the chemical industry, cosmetics, and many other fields.

Currently, the market demand for *P. sibiricum* is rising annually, which makes it challenging to satisfy societal demands solely through wild resource supplies. Thus, artificial cultivation has emerged as the primary solution to address the shortage of *P. sibiricum* resources. The propagation strategies of *P. sibiricum* include rhizome and seed reproduction. Due to the dormancy period of *P. sibiricum* seeds, the emergence cycle is prolonged, and the survival rate is low, which translates to high costs for large-scale artificial cultivation. Addressing the issue of lengthy dormancy has become crucial for the development of the *P. sibiricum* industry. The lignification of certain seed coat cells in *P. sibiricum* seeds results in poor permeability, which subsequently affects water absorption and respiratory metabolism. Further, the inadequate utilization of endosperm nutrients by *P. sibiricum* seeds amplifies the effects of internal inhibitors [7]. One of the factors that contributes to the dormancy of *P. sibiricum* is the presence of a post-maturity phenomena in the embryo and elevated ABA content [8]. Various inhibitors are present in the seed coats, endosperms, and embryos of *P. sibiricum*, with the highest concentrations found in the seed coats. These inhibitors maintain high activities across all components [9]. The dormancy attributes of *P. sibiricum* serve as critical mechanisms for its adaptation to the environment [10]. Research into the mechanisms behind endosperm weakening during *P. sibiricum* seed germination revealed that the endosperm contains sclereid-like cells. These cells exhibit characteristics such as thickened cell walls and the absence of protoplasts. During the endosperm weakening process, which occurs as the seeds develop, its cell walls become gradually thinner until they are completely degraded [11].

Investigations aimed at breaking the dormancy of *P. sibiricum* seeds indicated that 20 min ultrasonic treatments significantly increased their germination rates. This suggested that ultrasonic treatments enhanced amylase activities and seed permeability, thereby facilitating dormancy release [12]. The optimal germination effect was achieved at 4 °C when the seeds were layered with a mixture of river sand and frog rock at a 1:1 volume ratio. In contrast, treatments that involved strong acids, alkalis, and mechanical techniques were detrimental to seed germination [7]. The highest *P. sibiricum* seed germination rate was achieved under dark conditions using 150 mg/L GA_3_ and 100 mg/L 6-BA, after soaking the seeds at 40 °C for 24–30 h, and subsequently storing them at 0 °C for 120 d [13]. In summary, phytohormone treatments, warm water soaking, ultrasonic treatments, and layering treatments can effectively break the dormancy of *P. sibiricum* seeds. In contrast, chemical strategies such as the use of strong acids and alkalis, as well as physical approaches (e.g., the mechanical destruction of the seed coat) have adverse effects on dormancy breaking.

Despite the wealth of insights gained into the molecular regulatory mechanisms governing seed germination in a broad range of plant species, research efforts that couple transcriptomic and metabolomic approaches to elucidate the spatiotemporal dynamics of functional and bioactive compounds during *P. sibiricum* seed germination, as well as the intrinsic molecular regulatory mechanisms driving these processes, are still relatively limited and fragmented. Based on the observed changes in the content of key biochemical components in *P. sibiricum* seeds during sand storage, this study identified the critical factors restricting seed germination, and the findings provide novel technical approaches for the large-scale artificial cultivation of *P. sibiricum*.

Notably, this study’s innovative approach of using Huangjiang slag as a germination substrate for *P. sibiricum* seeds represents a threefold core innovation: it addresses the environmental challenge of industrial waste pollution by converting waste into a valuable resource; its unique carbohydrate composition and loose structure are leveraged to precisely meet the requirements of *P. sibiricum* seed germination for a sustained carbon source and a favorable microenvironment; and it establishes a circular ecological model of “industrial waste-to-medicinal plant cultivation” by replacing traditional peat substrates, reducing wetland destruction and dependence on chemical fertilizers, thereby providing a low-cost and sustainable solution for large-scale, eco-friendly cultivation of *P. sibiricum*.

## Materials and methods

### Plant materials

In this study, wild seeds of *P. sibiricum* were collected by the National Engineering Laboratory for Resource Developing of Endangered Chinese Crude Drugs in Northwest China (Shaanxi Normal University) from Lueyang County (33°31′25″ N, 106°11′38″ E, 955.4 m altitude), Hanzhong City, Shaanxi Province, China. The species was formally identified by Professor Kang Jiefang from Shaanxi Normal University, who was present at the collection site and confirmed the identification based on the morphological characteristics of the intact wild plants. The mature seeds were separated from the fruit, rinsed under running water, and air-dried at room temperature (22 ± 1 °C).To ensure consistency in seed dormancy status, uniform maturity criteria were adopted for seed selection in this study: fully mature, purple-black berries; plump seeds with a hard and lustrous testa; seed moisture content controlled within 9.0% ± 0.5%; and a thousand-seed weight of 32.5 ± 1.2 g. All these seeds, comprising over 1000 mature seeds, were collected from 50 individual plants within a single wild population. The seeds were thoroughly mixed and then divided into six equal aliquots, each containing approximately 150 seeds. Each aliquot underwent stratification and germination induction separately following an identical experimental protocol. For transcriptome analysis, three batches were randomly selected from the six aliquots for RNA extraction and sequencing to ensure comparability with the analytical results from the metabolomic samples. Following the completion of the experiments, the remaining seed material was stored at -80 °C.

### Optimal sand storage for germination

Response surface methodology (RSM) based on Box–Behnken design (BBD) was used to optimize the germination conditions of *P. sibiricum* seeds. Three factors at three levels were investigated: GA_3_ concentration (50, 125, and 200 mg/L; Factor A), sand storage temperature (− 4, 4, and 12 ℃; Factor B), and sand storage substrate (pure sand, 1/2 sand + 1/2 Huangjing slag, and 1/2 sand + 1/2 nutrient soil; Factor C). Factor C (substrate) was treated as a nominal categorical variable and excluded from quadratic terms in the model. Dummy variable coding was applied for regression analysis: pure sand was set as the reference group, with two dummy variables (C_1_ = 1 and C_2_ = 0 represent pure sand; C_1_ = 0 and C_2_ = 1 represent 1/2 sand + 1/2 Huangjiang slag; C_1_ = 1 and C_2_ = 1 represent 1/2 sand + 1/2 nutrient soil). Multiple regression analysis was carried out using Design-Expert 11 software to establish a model with germination rate as the response. Categorical variables were only included in main effects and interaction terms (no quadratic terms), which conforms to the statistical requirements of RSM (Table 1).

**Table 1:** The range and corresponding levels of the independent variables

Variables	Coded Factor Level		
	A	B	C
a, GA_3_ Concentration (mg/L)	50	125	200
b, Temperature (℃)	-4	4	12
c, Substrate	pure sand	1/2 sand + 1/2 Huangjiang slag	1/2 sand + 1/2 nutrient soil

### Observation of seed morphology

At the conclusion of the sand storage period, the seeds from each treatment group were randomly selected and longitudinally cut along the cotyledon axis to preserve the portion that contained the most embryos. Seeds sampled at days 0, 15, 30, 45, 60, 75, 90, and 105 were then observed using both stereomicroscopy and scanning electron microscopy.

### Endogenous phytohormone content detection

The 30-day sampling interval was adopted for the early stage of the experiment, which was determined based on the results of our pre-experiments; considering the relatively long seed germination cycle of *P. sibiricum*, this pre-validated interval is sufficient to accurately capture the phased dynamic variation trend of key biochemical components in seeds, and meanwhile avoids redundant sampling and the waste of experimental resources. The sampling interval was shortened to 15 days for the final sampling (from 90 d to 105 d) simply because the majority of seeds were observed to have completed germination at 105 d. Therefore, in this study, five stages with distinct embryonic development characteristics during seed germination were selected for endogenous phytohormone detection, transcriptomic, and metabolomic analyses, namely stage A (0 d), stage B (30 d), stage C (60 d), stage D (90 d), and stage E (105 d).

At 0, 30, 60, 90, and 105 days of seed germination, the levels of five endogenous phytohormones (ABA, GA_3_, JA, CTK, IAA) were quantified using enzyme-linked immunosorbent assay (ELISA) kits purchased from Shanghai Enzyme-linked Biotechnology Co., Ltd., with catalog numbers ml037429 (ABA), ml037433 (GA_3_), ml037448 (JA), ml037452 (CTK), and ml037425 (IAA). All kits exhibited high specificity, with cross-reactivity data specified in the product manuals showing 100% cross-reactivity with their target hormones and less than 0.1% cross-reactivity with the other four phytohormones tested, effectively avoiding mutual interference. Three biological replicates were set for each sampling time point during germination. The experimental data were statistically analyzed using DPS software and further visualized and processed with ORIGIN software.

### Total RNA extraction, cDNA library construction, and sequencing

Total RNA was extracted from *P. sibiricum* seeds at five germination stages (0, 30, 60, 90, and 105 days, designated as stages A, B, C, D, and E, respectively) using the TRIzol reagent. RNA integrity was precisely assessed using the Agilent 2100 Bioanalyzer, where detection scores ranging from 0 to 10 directly reflected RNA quality and integrity (higher scores indicated better quality). RNA-seq library construction, sequencing, and data analysis were performed by Personalbio Technology (Shanghai, China). The constructed libraries were sequenced on the Illumina platform, which utilizes unique bridge PCR amplification, with the flow cell automatically undergoing extension and imaging on the HiSeq 2500 platform. Image data generated from sequencing were converted to sequence data (reads) using CASAVA base calling. Raw data were preprocessed with fastp (version 0.19.7) using the following parameters: -g -q5 -u50 -n15 -l150. Additional filtering steps included removing sequences with 3’-end adapters using Cutadapt and discarding reads with an average quality score below Q20. After filtering, sequencing error rates were checked and GC content distribution was analyzed to obtain clean reads for subsequent analysis.

Since no reference genome is available for *P. sibiricum*, de novo assembly of the transcriptome was performed. Clean reads from all samples were pooled and assembled using Trinity, a de novo transcriptome assembler that reconstructs transcripts based on the De Bruijn graph (DBG) algorithm.

### Unigene annotation

After transcript assembly, unigenes were annotated using multiple databases including Pfam, SUPERFAMILY, Gene Ontology (GO), Kyoto Encyclopedia of Genes and Genomes (KEGG), NR, Swiss-Prot, KOG, and eggNOG. Specifically, blastx software was used to align the obtained sequences with NR, Swiss-Prot, KEGG, and KOG protein databases for functional annotation of proteins.

### Gene expression quantification and differential expression analysis

Gene expression levels were quantified as Fragments Per Kilobase of transcript per Million mapped reads to eliminate the influence of sequencing depth and gene length. Fragments per kilobase of transcript per million mapped reads (FPKM), a key metric for transcript quantification, is calculated using the following formula:

$$\mathrm{FPKM}=\frac{\mathrm{total}\;\mathrm{exon}\;\mathrm{Fragments}}{\mathrm{Mapped}\;\mathrm{reads}\left(\mathrm{Millions}\right)\ast\mathrm{exon}\;\mathrm{length}\left(\mathrm{KB}\right)}$$

Differential expression analysis between consecutive germination stages (A/B, B/C, C/D, D/E) was performed using DESeq2 (v1.20.0) software with raw count data. The Benjamini & Hochberg method was applied to correct p-values, and significantly DEGs were identified with the criteria of false discovery rate (FDR) < 0.05 and |log_2_ fold change (FC)| > 1.

### Functional enrichment analysis of DEGs

DEGs were subjected to GO, KEGG, STEM, and COG enrichment analyses. GO enrichment analysis categorized DEGs into biological process, cellular component, and molecular function categories. KEGG pathway enrichment analysis was conducted to identify significantly enriched metabolic pathways and signal transduction pathways using hypergeometric distribution tests.

### Screening of seed germination-related key genes

Based on functional annotations and enrichment results, DEGs related to seed germination were screened using the Swiss-Prot protein database. These key genes were classified according to their biological functions, and their expression patterns across different germination stages were analyzed.

### Quantitative real-time PCR analysis

To verify the reliability of the transcriptome sequencing data, the *TUB* gene of *P. sibiricum* was selected as an internal reference. The gene expression was detected via real-time fluorescence quantitative PCR (qRT-PCR) using cDNA from different germination stages of *P. sibiricum* seeds as templates. The 20 µL qRT-PCR reaction system (Table S1) included SYBR Premix Ex Taq™ II, gene-specific primers, RNase-Free H_2_O, and cDNA template, while the reaction procedure (Table S2) consisted of pre-denaturation, 35 cycles of denaturation-annealing-extension, and final extension. The specific primer sequences are provided in Table S3.

### Sample extraction and metabolite profiling

For *P. sibiricum* seeds at five germination stages (0, 30, 60, 90, and 105 days), 0.1 g of seed sample was accurately weighed from each biological replicate and mixed with 600 µL of methanol containing 4 ppm 2-chloro-L-phenylalanine (internal standard). The mixture was transferred to a 2 mL centrifuge tube, vortex-shaken for 30 s, and then ground in an ultrasonic grinder at 60 Hz for 90 s. Subsequently, ultrasonic extraction was performed at 40 Hz for 15 min, followed by centrifugation at 12,000 rpm and 4 °C for 10 min. The supernatant was collected and filtered through a 0.22 μm organic phase filter membrane, and the filtrate was transferred to a detection vial for LC-MS analysis.

The LC system used was a Thermo Vanquish (Thermo Fisher Scientific, USA), equipped with an ACQUITY UPLC^®^ HSS T3 column (2.1 mm × 150 mm, 1.8 μm; Waters, Milford, MA, USA). The injection volume was 2 µL, the column temperature was maintained at 40 °C, and the flow rate was set to 0.25 mL/min.

Two ion modes were employed for detection: For the positive ion mode, mobile phase C (0.1% formic acid in acetonitrile) and mobile phase D (0.1% formic acid in water) were used with the following gradient elution program: 0–1 min, 2% C; 1–9 min, 2%–50% C; 9–12 min, 50%–98% C; 12–13.5 min, 98% C; 13.5–14 min, 98%–2% C; 14–20 min, 2% C. For the negative ion mode, mobile phase A (acetonitrile) and mobile phase B (5 mM ammonium formate aqueous solution) were used with the following gradient program: 0–1 min, 2% A; 1–9 min, 2%–50% A; 9–12 min, 50%–98% A; 12–13.5 min, 98% A; 13.5–14 min, 98%–2% A; 14–17 min, 2% A.

Mass spectrometry was performed on a Thermo Q Exactive HF-X mass spectrometer (Thermo Fisher Scientific, USA) equipped with an electrospray ionization (ESI) source operating in both positive and negative ion modes. The ionization parameters were set as follows: spray voltage of 3.50 kV in positive mode and − 2.50 kV in negative mode; capillary temperature of 325 °C; sheath gas flow rate of 30 arb; auxiliary gas flow rate of 10 arb. Full-scan MS data were acquired at a resolution of 60,000 over an m/z range of 81–1000. For MS/MS fragmentation, data-dependent acquisition (DDA) was performed using higher-energy collisional dissociation (HCD) at a resolution of 15,000 with a normalized collision energy of 30%. The top eight most abundant ions were selected for fragmentation, with dynamic exclusion enabled to avoid redundant sequencing.

Metabolites were identified according to strict criteria including mass accuracy with a mass error < 30 ppm, matching of MS/MS spectra with reference spectra in standard or public databases (e.g., HMDB, MassBank, mzCloud), and retention time tolerance within ± 0.2 min.

To ensure data quality and minimize systematic errors, pooled quality control (QC) samples were prepared by mixing equal aliquots of all test samples and injected at regular intervals throughout the analytical run. A LOESS signal correction method based on QC samples was applied to the raw data to correct for systematic signal drift. Metabolite features with a relative standard deviation (RSD) exceeding 30% in QC samples were filtered out from subsequent analysis, ensuring that only stable and reliably detected ions were retained. The consistent peak intensity of the internal standard (2-chloro-L-phenylalanine) across all QC samples and test samples further indicated good extraction efficiency and instrumental stability throughout the entire analytical process.

### Statistical analysis of metabolomic data

Non-targeted metabolomic analysis was conducted by Metabo-Profile Biotechnology (Shanghai, China). Raw mass spectrometry data were converted to mzXML format using the MSConvert tool in the ProteoWizard software package. Peak detection, filtering, and alignment were performed using the XCMS software package with the following parameters: bw = 2, ppm = 15, peakwidth = c(5,30), mzwid = 0.015, mzdiff = 0.01, and method = centWave. Metabolite identification was conducted by matching with databases including HMDB, MassBank, LipidMaps, mzCloud, and KEGG, with a mass error threshold of ppm < 30.

To ensure comparability across samples and developmental stages, the obtained peak intensity matrix was first normalized using the total ion current (TIC) method to correct for variations in sample injection and ionization efficiency. Subsequently, to enhance the reliability and interpretability of multivariate pattern recognition analyses, the normalized data were autoscaled. This process mean-centers each metabolite feature and scales it by its standard deviation, thereby giving each metabolite equal weight in the models and providing more robust and intuitive results for distinguishing between different germination stages.

Multivariate statistical analyses were performed using the R software package Ropls, including principal component analysis (PCA), and orthogonal partial least squares discriminant analysis (OPLS-DA). A permutation test was conducted to evaluate the stability of the OPLS-DA model and avoid overfitting. Differential metabolites were screened based on the criteria of p-value < 0.05 (from t-test) and variable importance in projection (VIP) > 1 (calculated from OPLS-DA). Functional pathway enrichment and topological analysis of differential metabolites were performed using the MetaboAnalyst software package.

### Experimental design of biological replicates

Six independent biological replicates were set for each germination stage to ensure the reliability and reproducibility of metabolomic data. These replicates were derived from independent seed batches collected from different individual plants within the same *P. sibiricum* population. Each replicate underwent separate sand stratification treatment, independent sampling, and independent extraction procedures, with no cross-contamination or shared processing steps between replicates. This experimental design minimized sampling bias and effectively captured the inherent biological variability.

## Results

### Response surface optimization analysis

#### Model fitting and statistical analysis

A total of 17 experiments were conducted, and the specific parameter combinations along with the corresponding germination rates of *P. sibiricum* seeds are shown in Table 2. Under different parameter combinations, the actual germination rates ranged from 56.2% to 89.65%. Multiple regression analysis of the experimental data yielded a second-order polynomial equation describing the relationship between germination rate and the independent variables as follows:

$$\begin{aligned} \mathrm{Germination}\;\mathrm{rate}=&\:82.6993+0.07442\times\mathrm{A}+1.2775\times\mathrm{B}-6.5761\times\mathrm{C}_{1}\\&+6.6947\times\mathrm{C}_{2}-3.1575\times\mathrm{AB}-2.8742\times\mathrm{AC}_{1}\\&+1.3733\times\mathrm{AC}_{2}+0.9525\times\mathrm{BC}_{1}+1.855\times\mathrm{BC}_{2}\\&-11.9845\times\mathrm{A}^{2}-12.802\times\mathrm{B}^{2} \end{aligned}$$

where A is GA_3_ concentration; B is sand storage temperature; C_1_ = 1 and C_2_ = 0 represent pure sand; C_1_ = 0 and C_2_ = 1 represent 1/2 sand + 1/2 Huangjiang slag; C_1_ = 1 and C_2_ = 1 represent 1/2 sand + 1/2 nutrient soil.

**Table 2:** The Box-Behnken design with response values for Germination conditions

Run	a (mg/L)	b (℃)	c	Germination rate (%)	
				Experimental	Predicted
1	50	-4	2	56.2	58.62
2	125	12	3	68.1	69.64
3	200	4	1	61.86	63.04
4	50	12	2	68.78	68.42
5	200	-4	2	66.75	67.11
6	125	4	2	88.97	89.39
7	125	-4	3	71.16	69.92
8	125	4	2	89.65	89.39
9	125	12	1	65.7	66.94
10	50	4	3	68.5	67.32
11	125	4	2	89.31	89.39
12	200	4	3	72.99	73.87
13	125	-4	1	61.24	59.7
14	125	4	2	89.59	89.39
15	200	12	2	66.7	64.28
16	50	4	1	66.12	65.24
17	125	4	2	89.45	89.39

Furthermore, the analysis of variance (ANOVA) for the model is presented in Table S4. The model F-value was 2058.88 with a p-value < 0.0001, indicating that the model was significant. In this model, the p-values for A, B, C, AB, AC, BC, A^2^, and B^2^ were all less than 0.05, demonstrating that all terms had significant effects on germination rate. The coefficient of determination (R^2^) was 0.9998, and the adjusted R^2^ was 0.9993, indicating that the model explained 99.98% of the response variable variation and exhibited excellent fit. The coefficient of variation (C.V.%) was 0.4204, which is far below 10%, suggesting high experimental precision and good reproducibility. The adequate precision (Adeq Precision) was 128.74, which is much higher than the critical value of 4, indicating that the model possesses strong predictive capability and can be reliably used for optimization and prediction.

### Response surface analysis

**Fig. 1:**
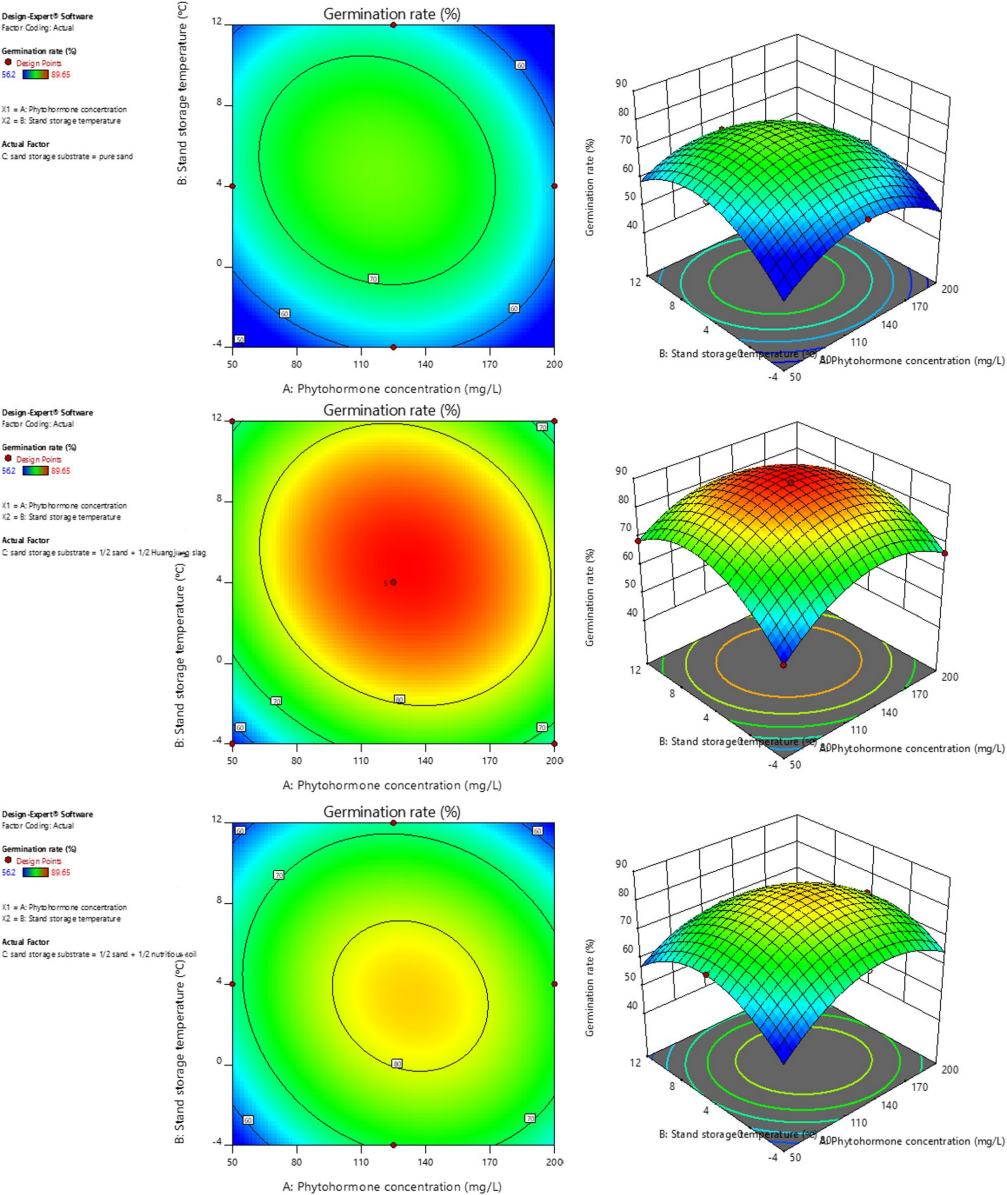
3D response surface and 2D contour map of the response surface model for *P. sibiricum* seed germination optimization. Figure legend: This figure shows the three-dimensional response surface plot (left) and two-dimensional contour plot (right) for optimizing the germination conditions of *P. sibiricum* seeds using the response surface methodology (BBD design). The independent variables include: **A** = GA_3_ concentration (mg/L), **B** = stratification temperature (°C), with the response value being the seed germination rate (%). The 3D response surface reflects the nonlinear relationship between each independent variable and the germination rate, while the elliptical shape of the 2D contour plots indicates significant interactions among the independent variables (consistent with the ANOVA results in Table S4: model p < 0.0001, R^2^ = 0.9998)

As shown in Fig. 1, the relationship between germination rate and the three independent variables was analyzed by constructing three-dimensional response surface plots and two-dimensional contour plots. The contour plots were all elliptical in shape, indicating a significant interaction between GA_3_ concentration and sand storage temperature on germination rate, which was consistent with the ANOVA results. The figure also shows that germination rate was significantly higher under the substrate condition of 1/2 sand + 1/2 Huangjiang slag.

Additionally, the optimal germination conditions were predicted as follows: a GA_3_ concentration of 130.497 mg/L, sand storage temperature of 4.90 °C, and sand storage substrate of 1/2 sand + 1/2 Huangjiang slag. Under these predicted conditions, the model indicated a theoretical maximum germination rate of 89.65% ± 0.31%. Experimental validation under the predicted optimal conditions produced a germination rate of 89.31% ± 0.34%, demonstrating the robustness of the optimized parameters.

### Seed morphology observation 

#### Morphological changes under stereomicroscopy

**Fig. 2:**
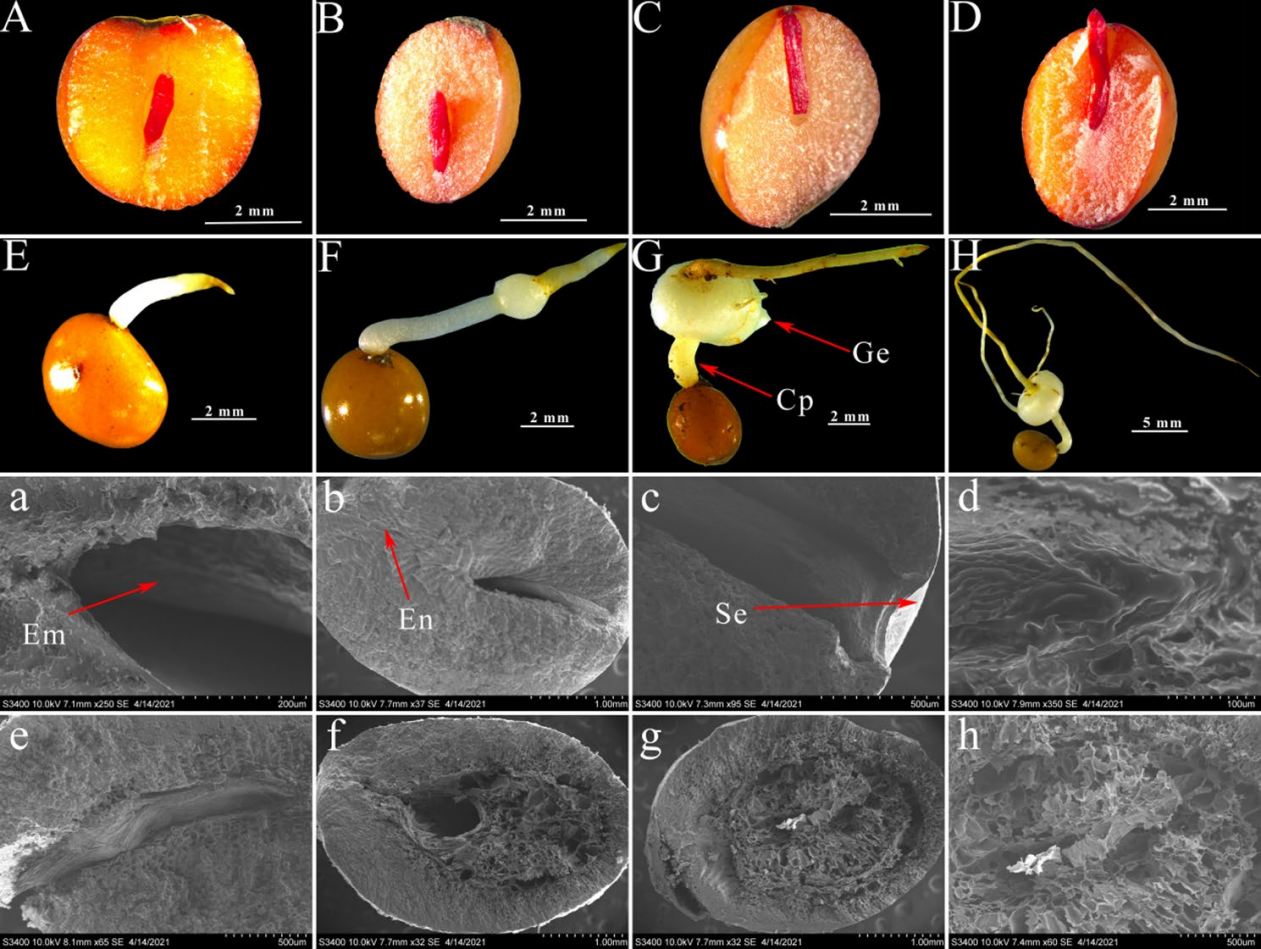
Morphological changes during sand storage and germination of *P. sibiricum* seeds. Figure Legend: Stereomicroscopy observations, **a**–**h** Corresponding scanning electron microscopy of vertical cross-sections (0–105 d of sand storage). **A**–**D** (**a**–**d**): Dormancy and embryo development stage with embryo elongation and endosperm weakening; **E**–**H** (**e**–**h**): Post-germination rhizome and bud formation stage. Red arrows point to key structural features. Abbreviations: Em = Embryo, En = Endosperm, Sc = Seed coat, Ge = Germ, Cp = Connecting points

Seeds stored in sand for 0, 15, 30, 45, 60, 75, 90, and 105 days were selected for morphological observation (Fig. 2, A-H). The seeds were cut vertically along the cotyledon axis, retaining more than half of the embryos. Stages E to H represent the rhizome processes following seed germination. In stage A, the seeds are dormant and the embryo is small, measuring only one-third the length of the vertical axis. As the seeds absorb water, the weakening of the endosperm and the metabolism of various nutrients drive embryo growth. At 15 days, the elongation of the embryo was clearly evident. By 30 days, the embryos had approached the seed coat, accompanied by the loosening of the cell wall and weakening of the endosperm. Between 45 and 60 days, the seed embryo broke through the seed coat to complete germination. Subsequently, the embryo continued to expand to form a spherical stem and sprout (E-H).

#### Morphological changes observed by scanning electron microscope

Using scanning electron microscopy, the weakening process of the endosperm could be clearly observed (Fig. 2). Panels A-H correspond to the sequential stages of endosperm weakening, with stages a-h representing their respective magnified views. At stages a and b, the endosperm appeared dense and firm, which facilitated continuous germination. By stage c the endosperm exhibited a weak honeycomb structure, a feature that became more pronounced in stage d. By this point, the seed had completed germination. As the embryo expanded further, most of the endosperm was degraded by stage h. This degradation not only supplied essential energy resources for embryo growth but also alleviated physical constraints on the embryo, which enabled easier elongation.

#### Changes in endogenous phytohormones

**Fig. 3:**
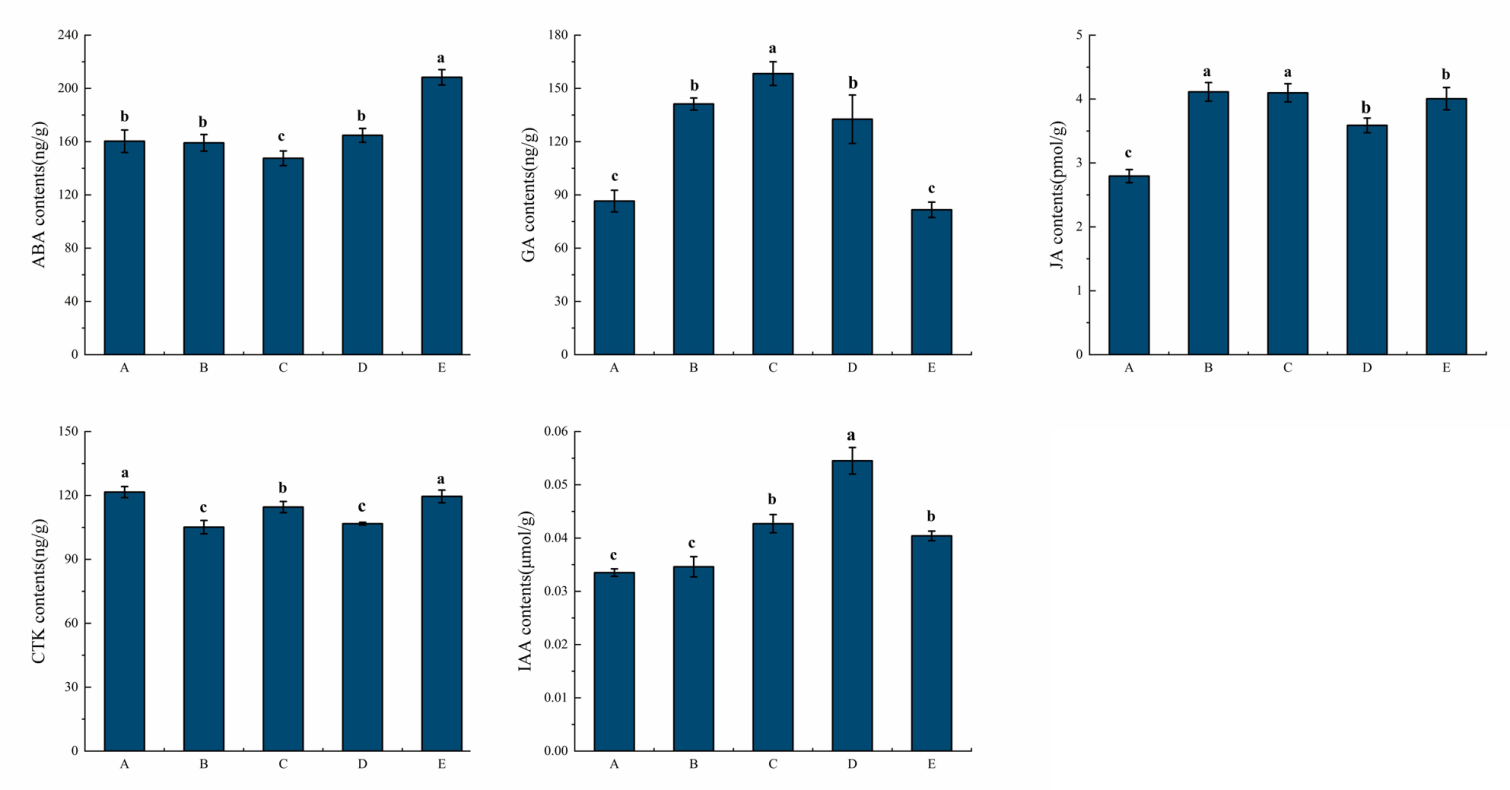
Changes of endogenous phytohormones during germination of seeds of *P. sibiricum* seeds (A: 0 d, B: 30 d, C: 60 d, D: 90 d, E: 105 d)

During the germination of *P. sibiricum* seeds, ABA content showed a trend of first decreasing then increasing, declining at stages B and C to the lowest point at stage C (embryo just breaking through the seed coat for germination completion), rebounding at stage D and peaking at stage E, ABA decreasing first and then increasing during seed germination may be closely related to the survival of the seedlings [14]. GA_3_ content was low in dormant seeds, rose rapidly with dormancy release and germination progress to a maximum at stage C (twice the level of stage A), and then decreased to a normal level during subsequent radicle and spherical stem development. JA content had a similar first-increasing then-decreasing trend to GA_3_, being low in dormancy, rising with germination with comparable levels at stages B and C, slightly declining at stage D and rebounding at stage E, and its distinct feature was maintaining a relatively high level both before and after germination compared with the dormancy period. CTK content showed no obvious regular pattern throughout the germination process, while IAA content had a clear variation trend: it was the lowest in dormancy, rose to the maximum at stage D with germination progress, and decreased after spherical stem formation (Fig. 3).

#### Quality of transcriptome data and unigene assembly

The RNA-seq analysis of five samples yielded a total of 42.4 Gb of clean data, with each sample contributing no less than 6 Gb. Detailed filtering statistics are provided in Table S6. On average, clean reads accounted for > 90% of raw reads, with Q20 exceeding 96%, Q30 surpassing 92%, and GC content averaging > 48%. These results confirmed that the sequencing data were of high quality and suitable for downstream analyses.

Notably, each germination stage (A, B, C, D, E) included 3 independent biological replicates for transcriptomic analysis (e.g., A1/A2/A3 for stage A; Table S5), which were derived from independently cultured seed groups of *P. sibiricum* to ensure experimental independence and reduce individual sampling bias. For transcriptomic data variability, the FPKM values of biological replicates were normalized using DESeq2 (v1.20.0) , and statistical significance of differential gene expression was determined by Benjamini & Hochberg method for multiple testing (FDR < 0.05), which effectively accounted for inherent variability among the three transcriptomic replicates. 

### Unigene annotation results

Unigene annotation results across different databases are summarized in Table 3. The NR database annotated 87,651 unigenes (46.38%), of which 36,542 were annotated to the seven species with the highest sequence similarity (accounting for 41.7% of total NR annotations), and 58.3% were annotated to other plant species (Table 3, Fig. S1). Functional annotation using the GO database categorized 34,070 unigenes (18.03%) into three main categories and 48 subcategories (Table 3, Fig. S2C). The KEGG database annotated 55,095 unigenes (29.15%), with the most frequently annotated pathways being translation, signal transduction, and carbohydrate metabolism (Fig. S2D). Additionally, 86,903 unigenes (45.98%) were annotated in the Swiss-Prot database and further cross-validated by the UniProtKB database, while 83,360 unigenes (44.11%) were successfully annotated in the eggNOG database, with the top functional categories including general function prediction, unknown function, and post-translational modification (Fig. S2E). A total of 20,442 unigenes (10.82%) were annotated across all databases.

**Table 3:** Summary of annotation results

Database	Number	Percentage
NR	87,651	46.38
GO	34,070	18.03
KEGG	55,095	29.15
Pfam	73,433	38.86
eggnog	83,360	44.11
Swiss-Prot	86,903	45.98
In all databases	20,442	10.82

*Number*  number of successfully annotated unigenes, * Percentage * proportion of successfully annotated unigenes to total unigenes

### Different DEGs 

**Fig. 4:**
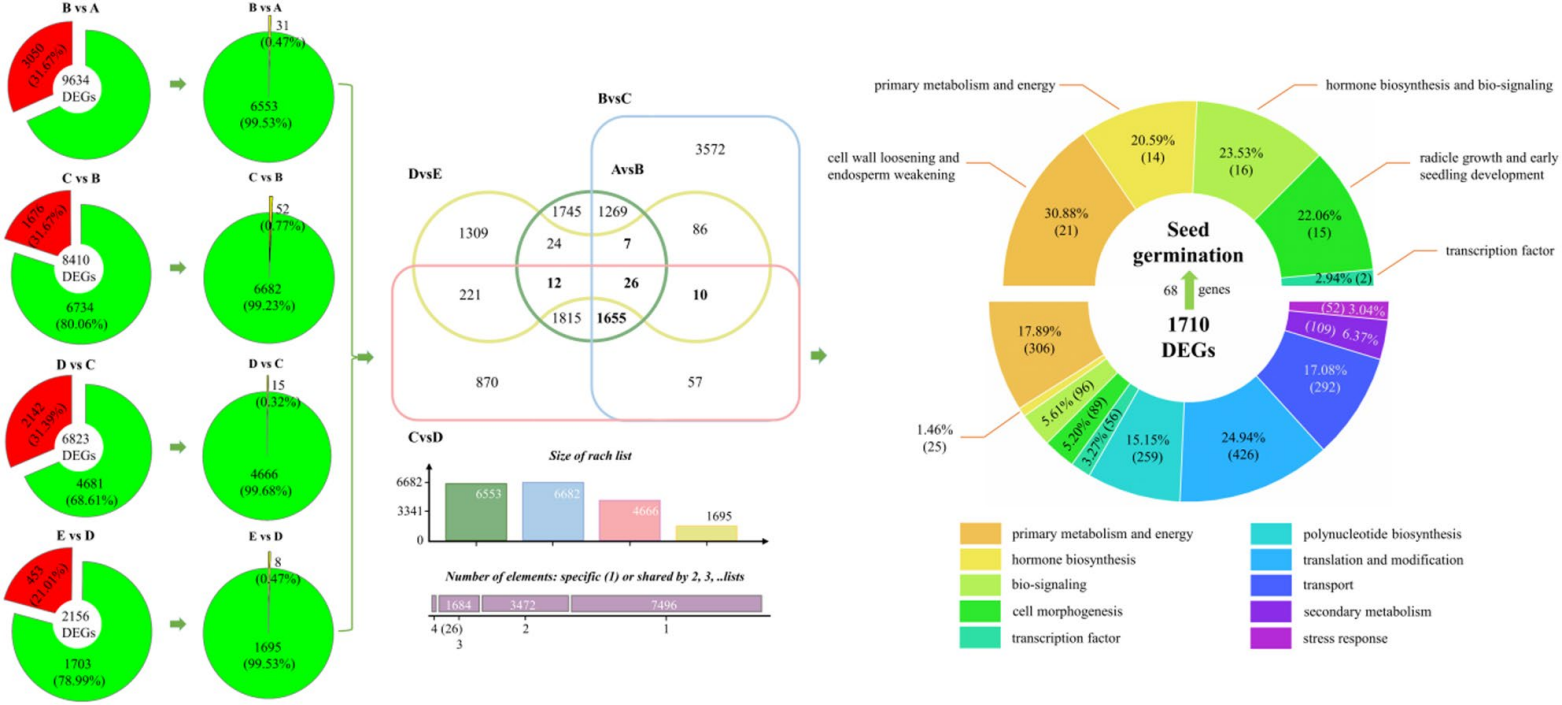
Distribution and classification of differentially expressed genes (DEGs) in *P. sibiricum* seeds at different germination periods (A: 0 d, B: 30 d, C: 60 d, D: 90 d, E: 105 d)

Gene expression analyses across different germination stages revealed 9,634, 8,410, 6,823, and 2,156 significant DEGs in stages A/B, B/C, C/D, and D/E, respectively (Fig. 4). Among these DEGs, 6,584, 6,734, 4,681, and 1,703 genes were annotated using GO, KOG, Swiss-Prot, and KEGG databases, respectively, and 6,553, 6,682, 4,666, and 1,695 of these annotated genes exhibited functional characteristics.

Furthermore, we identified 1,710 functionally annotated differentially expressed genes (DEGs) that were consistently differentially expressed across at least four pairwise comparisons between adjacent germination stages (B/A, C/B, D/C, and E/D). To ensure a clear and unambiguous functional annotation, we assigned each co-expressed DEG to only one primary functional category based on its most prominent biological role. These co-expressed DEGs were systematically classified into 10 functional categories based on their biological roles: primary metabolism and energy (306), phytohormone biosynthesis (25), biological signal transduction (96), cell morphogenesis (89), transcription factors (56), polynucleotide biosynthesis (259), translation (426), transport (292), secondary metabolism (109), and stress response (52) (Fig. 4). 

### Identification and expression patterns of seed germination-related key genes 

The 68 key genes related to seed germination were identified through a rigorous multi-step screening process and grouped into five functional categories: cell wall loosening and endosperm weakening (21), primary metabolism and energy (14), phytohormone biosynthesis and biological signal transduction (16), radicle growth and early seedling development (15), and transcription factors (2) (Fig. 4). Specifically, we first filtered for significantly differentially expressed genes (DEGs) with FDR < 0.05 and |log_2_ FC| > 1 to ensure statistically significant expression changes across germination stages; then, we annotated these DEGs using the Swiss-Prot protein database to retain only those with functional annotations directly relevant to seed germination, including cell wall modification, endosperm weakening, phytohormone regulation, primary metabolism, and radicle development; finally, we further refined the gene set by selecting those expressed in at least 4 out of 5 germination stages with an average FPKM value > 10, thereby excluding low-abundance and transiently expressed genes to ensure the reliability and biological relevance of the identified key genes.

**Fig. 5:**
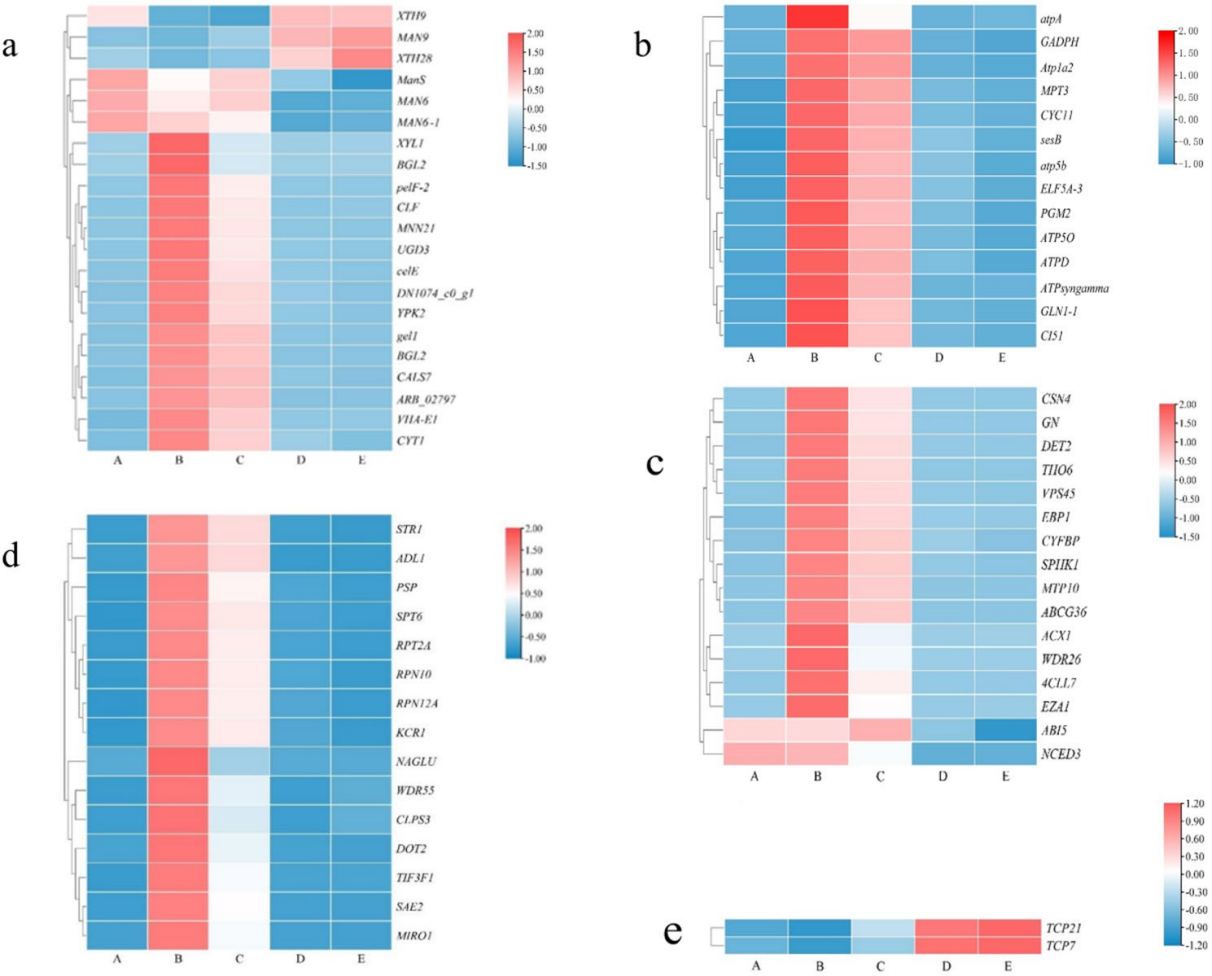
Expression patterns of key genes related to *P. sibiricum* seed germination (A: 0 d, B: 30 d, C: 60 d, D: 90 d, E: 105 d); **a** Expressions of genes related to cell wall loosening and endosperm weakening; **b** Expressions of genes related to primary metabolism; **c** Expressions of genes related to phytohormone synthesis and signaling; **d** Expressions of genes associated with radicle growth and early seedling development; **e** Expressions of genes related to transcription factors

Cell wall loosening and endosperm weakening-related genes: A total of 21 DEGs were identified, including *CALS-7*, *DN1074*, *VHA-E1*, *gel1*, *BGL2*, *XYL1*, *ARB*, *MNN21*, *BGL2-1*, *YPK2*, *celE*, *UGD3*, *pelF-2*, *CLF*, *MAN9*, *CYT1*, *XTH28*, *XTH9*, *ManS*, *MAN6*, and *MAN6-1*. *XTH9*, *MAN9*, and *XTH28* exhibited peak expressions during stages D and E, while *ManS*, *MAN6*, and *MAN6-1* showed the highest expression levels during stages A to C. The remaining 15 genes had minimal expressions in stage A but were significantly upregulated in stages B and C, with expression levels tapering off after germination completion in stages D and E (Fig. 5a).

Primary metabolism and energy-related genes: Fourteen genes (*atp5b*, *ELF5A-3*, *MPT3*, *GADPH*, *ATP5O*, *GLN1-1*, *ATPs*, *PGM2*, *sesB*, *ATPD*, *CI51*,* Atp1a2*, *atpA*, and *CYC11*) exhibited similar expression patterns, peaking during stages B and C, and played crucial roles in providing energy for seed germination (Fig. 5b).

Phytohormone biosynthesis and signal transduction-related genes: Sixteen genes (*EBP1*, *CYFBP*, *ACX1*, *SPHK1*, *MTP10*, *DET2*, *4CLL7*, *THO*, *CSN4*, *VPS45*, *GN*, *WDR26*, *EZA1*, *ABCG36*, *ABI5*, and *NCED3*) were involved in this category. *ABI5* and *NCED3* were prominently upregulated during stages A, B, and C but downregulated in stages D and E, reflecting their critical roles in hormone regulation (Fig. 5c).

Radicle growth and early seedling development-related genes: Fifteen genes (*RPT2*, *STR1*, *ADL1*, *RPN10*, *RPN12A*, *KCR1*, *PSP*, *SAE2*, *MIRO1*, *WDR55*, *DOT2*, *CLPS3*, *TIF3F1*, *NAGLU*, and *SPT6*) were predominantly upregulated during stages B and C, with lower expressions during stages A, D, and E (Fig. 5d).

Transcription factors: *TCP7* and *TCP21* were identified as key transcription factors, showing significant upregulation during stages D and E with peak expression in stage E, highlighting their pivotal regulatory roles in seed germination (Fig. 5e).

### Functional enrichment of DEGs

GO enrichment analysis showed that DEGs were mainly categorized into biological process (cellular processes, metabolic processes, response to stimuli, biological regulation), cellular component (protein-containing complexes, cellular anatomical entities), and molecular function (binding, catalytic activities) categories. KEGG pathway enrichment analysis revealed that DEGs were significantly enriched in metabolic pathways related to seed germination, including primary metabolism, energy metabolism, phytohormone biosynthesis, and cell wall metabolism, which were consistent with the functional classification of key genes. These results indicated that DEGs were extensively involved in regulating material metabolism, energy supply, hormone balance, and structural modification during *P. sibiricum* seed germination.

### Regulatory mechanism of DEGs in seed germination and radicle growth

**Fig. 6:**
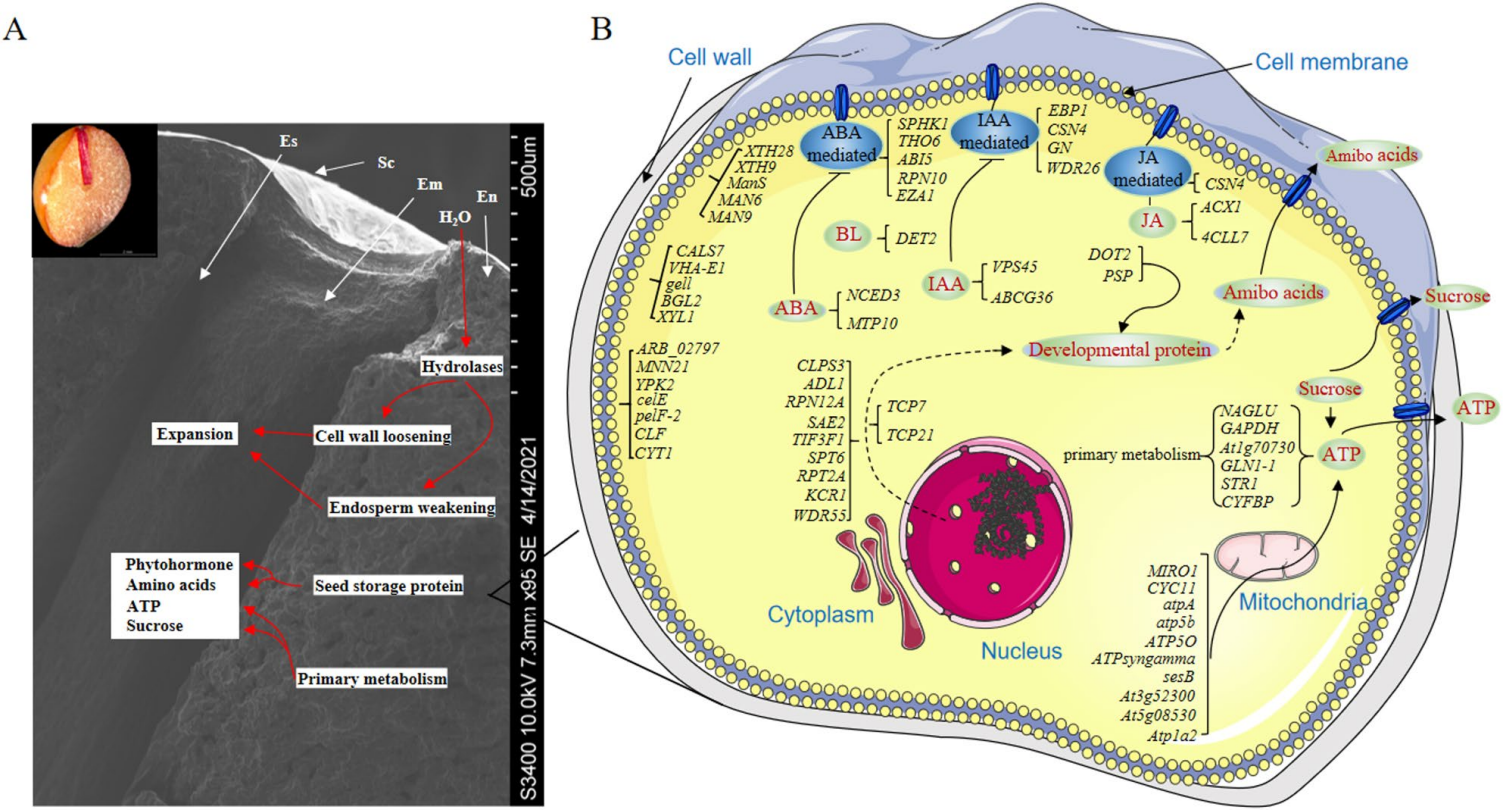
Gene regulatory network during P. sibiricum seed germination; **A** Scanning electron micrograph of P. sibiricum seed during germination; **B** Schematic diagram of the hormone-mediated gene regulatory network during seed germination.Figure Legend: White arrows point to key structural features. En=Endosperm, Em=Embryo, Sc=Seedcoat, Es= Endosperm surface

During *P. sibiricum* seed germination and bulb development, water absorption and seed expansion initiate the activation of hydrolases. This activation promotes endosperm weakening and cell wall loosening, thereby inducing embryo development. Concurrently, stored proteins and energy reserves (e.g., starch, lipids, and fatty acids) are degraded and metabolized into amino acids, sucrose, and ATP, which provide the essential energy for embryo elongation and subsequent seed coat breakage (Fig. 6A).

At the cellular level, DEGs associated with cell wall loosening and endosperm weakening (e.g., *CALS-7*, *DN1074*, *VHA-E1*, *gel1*, *BGL2*, *XYL1*, *ARB*, *MNN21*, *BGL2-1*, *YPK2*, *celE*, *UGD3*, *pelF-2*, *MAN9*, *CYT1*, *XTH28*, *XTH9*, *ManS*, *MAN6*, and *MAN6-1*) are predominantly localized near the cell wall. Among these, the *TCP* transcription factor family (*TCP7* and *TCP21*) localized in the nucleus induces the expression of genes such as *ADL1* and *SPT6*, which play crucial roles in promoting cell wall degradation and embryo development.

In the cytoplasm, genes including *NAGLU*, *GADPH*, *PGM2*, *GLN1-1*, *STR1*, and *CYFBP* facilitate the degradation and metabolism of nutrients such as starch and sugars. Meanwhile, mitochondrial genes (e.g., *MIRO1*, *atpA*, *atp5b*, *ATP5O*, *ATPsyngamma*, *sesB*, *ATPD*, *CI51*, *Atp1a2*, and *CYC11*) are involved in ATP synthesis and degradation, providing essential energy for embryo elongation.

Furthermore, genes such as *ABI5*, *GN*, and *ACX* participate in the synthesis and degradation of phytohormones (ABA, IAA, and JA), which regulate key signaling pathways underlying seed germination (Fig. 6B). Collectively, these DEGs form a coordinated regulatory network involving cell wall modification, nutrient metabolism, energy supply, and phytohormone balance to drive *P. sibiricum* seed germination and radicle growth. 

### qRT-PCR validation

A total of 10 genes were selected for qRT‑PCR validation to verify the reliability of the transcriptome data. These genes were chosen based on two key criteria: first, they represent distinct functional categories involved in seed germination, including phytohormone signaling (e.g., *NCED3*, *ABI5*), energy metabolism (e.g., *atpA*, *atp5b*, *ATP5O*), and transcriptional regulation (e.g., *TCP7*, *TCP21*), ensuring that the validation reflects global transcriptome changes; second, they exhibited significant differential expression in the RNA‑seq data, with high fold changes and stable expression patterns.

The qRT‑PCR results (Fig. S4) showed highly consistent expression trends with the RNA‑seq FPKM profiles across all five germination stages. Pearson correlation analysis revealed a strong positive correlation between the two datasets, with correlation coefficients (R) ranging from 0.6956 to 0.9987 for the 10 validated genes. Most genes displayed extremely high correlation (R > 0.90), and even the lowest R value (0.6956 for ABI5) remained within an acceptable range, further confirming the reliability and accuracy of our transcriptome sequencing results. 

### Quality control analysis of metabolic samples

After chromatographic separation and mass spectrometric detection, base peak chromatograms (BPCs) and total ion chromatograms (TICs) were generated to evaluate the stability of the LC-MS system and sample repeatability. The TICs of *P. sibiricum* seeds under positive and negative ion modes (Fig. S5A and S5B) showed consistent experimental trends across all samples, indicating good reproducibility of the detection system and suitability for subsequent analyses.

**Fig. 7:**
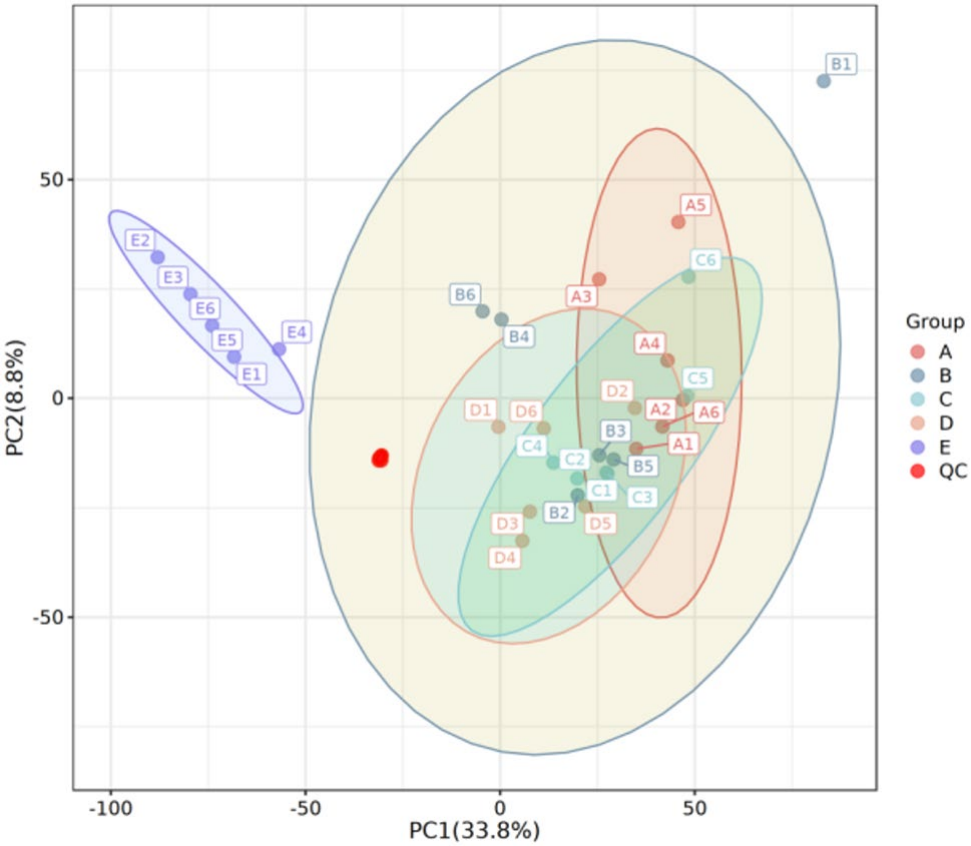
PCA scores of different stages of seed germination of *P. sibiricum* (A: 0 d, B: 30 d, C: 60 d, D: 90 d, E: 105 d)

PCA was performed to assess the overall separation trend and inter-replicate variability of samples. As shown in Fig. 7 , the six biological replicates of each germination stage clustered closely together, except for a slight deviation in sample B1 (which provided limited data reference). Clear separation was observed between different germination stages, confirming the reliability of the metabolomic data and the distinct differences in metabolite profiles among stages. This approach summarizes the characteristics of the metabolic profiles of samples and visually illustrates the separation trends between samples [15]. 

The OPLS-DA score plots (Fig. S6) showed that samples from the five germination stages were clearly separated and distributed within the 95% confidence interval, further verifying the significant differences in metabolite compositions among stages. The permutation test results (Fig. S7) indicated that all blue points (from permuted models) were lower than the original Q^2^ point, demonstrating that the OPLS-DA model was stable and free from overfitting. These results collectively confirmed that the metabolomic data were of high quality and suitable for subsequent differential metabolite screening. 

### Screening and quantification of differential metabolites

**Fig. 8:**
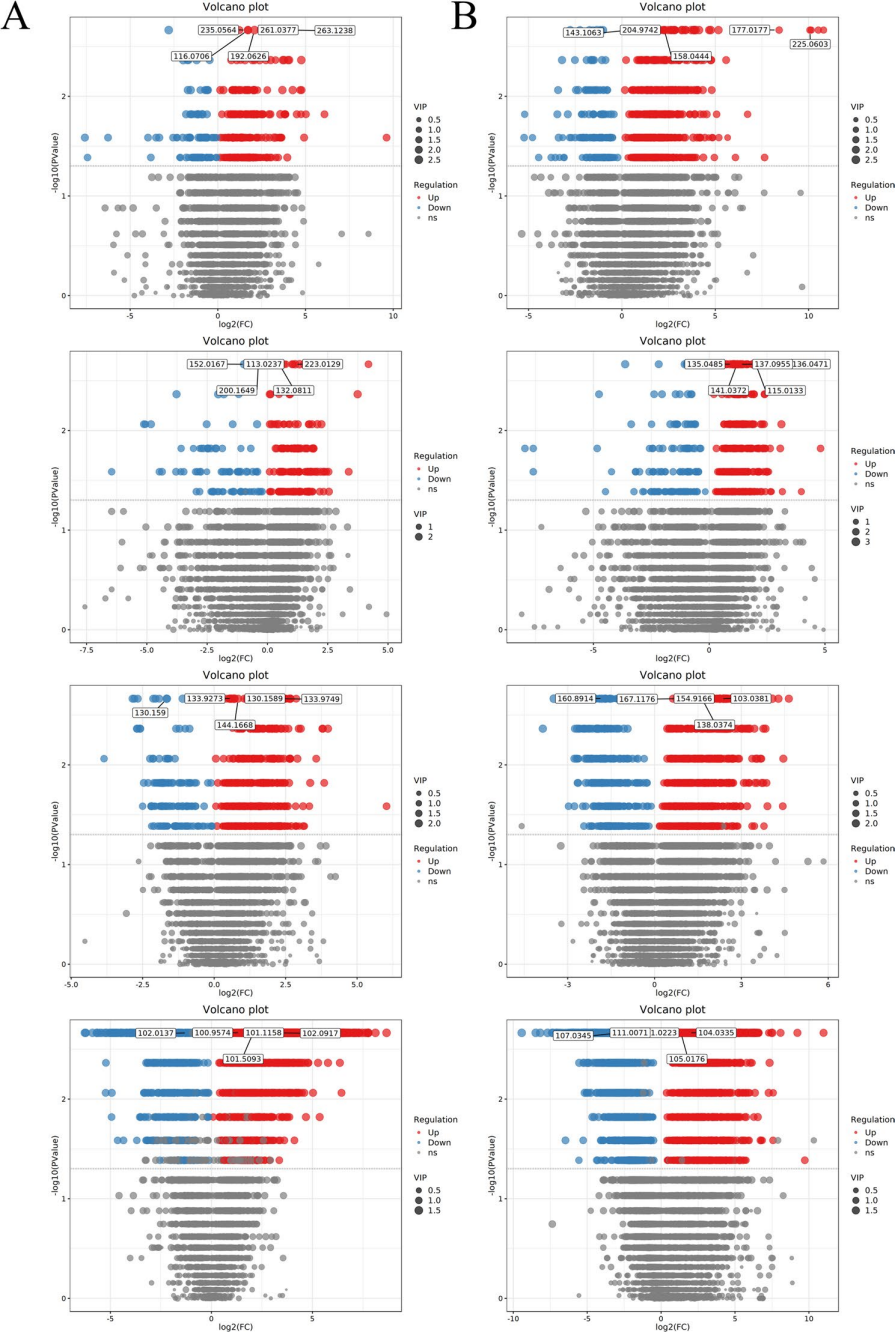
Volcano plots for differential metabolite screening (**A**: positive ion mode; **B**: negative ion mode). Figure legend: Volcano plots of differential metabolites in pairwise comparisons of *P. sibiricum* seed germination stages (from top to bottom: 0 d vs 30 d, 30 d vs 60 d, 60 d vs 90 d, 90 d vs 105 d)

Volcano plots were used to visually display the differences in metabolite abundance between consecutive germination stages (BvsA, CvsB, DvsC, EvsD) (Fig. 8). Each point in the volcano plot represents a metabolite, with point size indicating VIP value and color distinguishing upregulated (red) and downregulated (blue) metabolites. The x-axis represents log_2_-transformed FC of metabolite abundance, and the y-axis represents -log_10_-transformed p-value.

Under positive ion mode, the number of differential metabolites identified in each stage was as follows: 346 (249 upregulated, 97 downregulated) in BvsA; 262 (174 upregulated, 88 downregulated) in CvsB; 662 (526 upregulated, 136 downregulated) in DvsC; and 2458 (1980 upregulated, 478 downregulated) in EvsD. Notably, the dramatic surge in differential metabolites at the EvsD stage is biologically consistent with the late germination period: at this stage, the seed embryo completes elongation and transitions to post-germination seedling establishment, requiring large-scale metabolic reprogramming to support rapid cell division and tissue differentiation.

Under negative ion mode, a total of 798 differential metabolites were identified across all germination stages. For each consecutive stage under negative ion mode, the number of differential metabolites was: 635 upregulated and 157 downregulated in BvsA; 405 upregulated and 119 downregulated in CvsB; 817 upregulated and 369 downregulated in DvsC; and 1209 upregulated and 1820 downregulated in EvsD. Correspondingly, the significant increase in differential metabolites (especially downregulated lipid and storage sugar metabolites) at the EvsD stage in negative ion mode reflects the consumption of stored reserves (e.g., lipids and polysaccharides) to provide energy and carbon skeletons for the final transition from seed dormancy to autotrophic seedling growth. 

When integrating data from both positive and negative ion modes for differential metabolite screening, we employed a rigorous standard procedure to avoid redundancy and conflicts. For metabolites that were identified as the same compound in both ion modes and exhibited consistent trends (i.e., both up-regulated or both down-regulated), they were retained only once in the final list. In such cases, the Fold Change (FC) and p-value were derived from the ion mode that showed higher significance (i.e., a smaller p-value) or a larger absolute FC value in the integrated analysis.

If the qualitative or quantitative results for the same metabolite showed significant conflicts between the two modes—such as qualitative matching scores below the threshold or opposing expression trends—the metabolite was excluded to prevent erroneous data from interfering with subsequent analyses.

All screening and processing of duplicate metabolites were conducted in accordance with stringent metabolomics quality control standards (mass-to-charge ratio error < 30 ppm, retention time deviation ± 0.2 min).

**Fig. 9:**
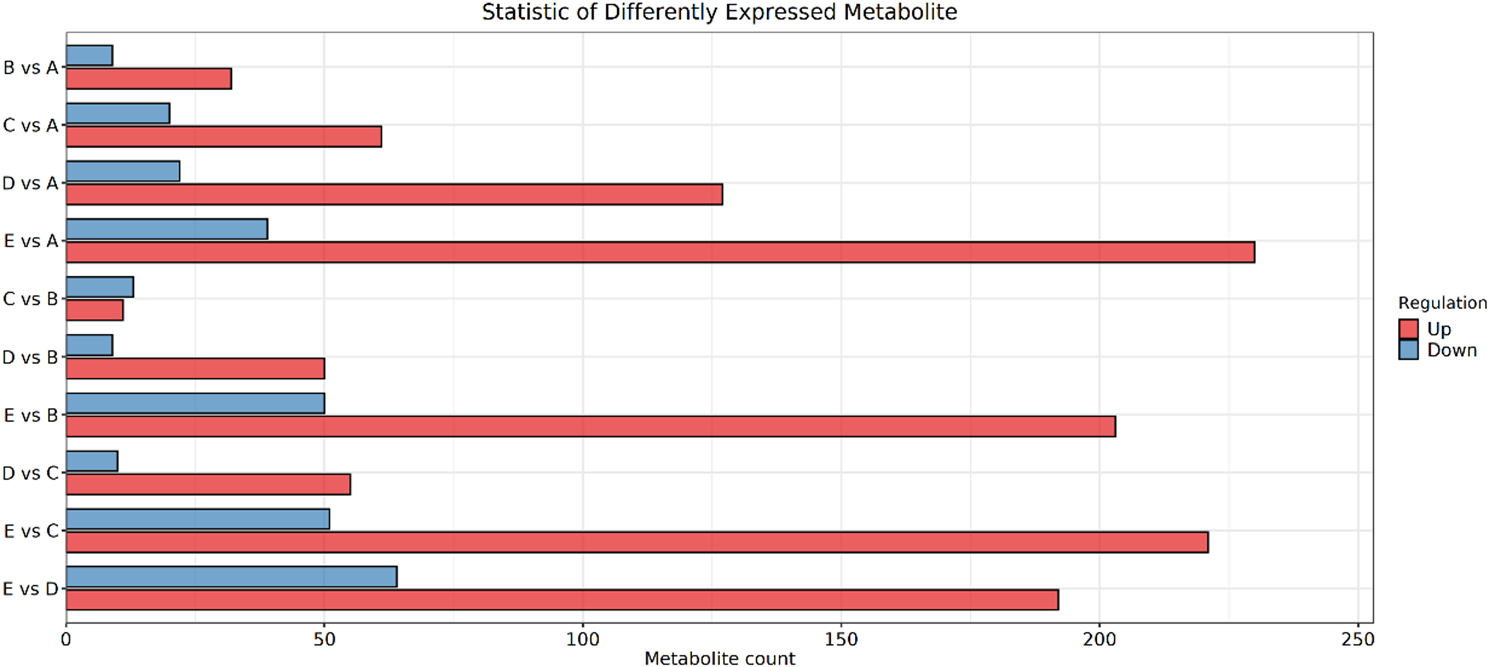
Statistics on the number of differential metabolites in positive and negative ion modes(A: 0 d, B: 30 d, C: 60 d, D: 90 d, E: 105 d)

When integrating the results of both ion modes, 41 differential metabolites were detected in BvsA (32 upregulated, 9 downregulated), 24 in CvsB (11 upregulated, 13 downregulated), 65 in DvsC (55 upregulated, 10 downregulated), and 256 in EvsD (192 upregulated, 64 downregulated) (Fig. 9). The number of differential metabolites increased significantly with the progression of germination, especially in the EvsD stage, indicating profound metabolic reprogramming during late germination. 

### Identification of key differential metabolites

To identify core metabolites involved in *P. sibiricum* seed germination, differential metabolites present across all five stages were screened using the criteria of p-value < 0.05 and FC > 2. A total of 26 key differential metabolites were identified, including 11 upregulated and 15 downregulated metabolites.

Upregulated metabolites included Ferreirin, (13E)-11α-Hydroxy-9,15-dioxoprost-13-enoic acid, Avermectin B1b aglycone, Berberine, dAMP, (S)-Reticuline, Methyl jasmonate, Homocitric acid, Sertraline, 4-(2-aminophenyl)-2,4-dioxobutanoic acid, and AMP. Downregulated metabolites included Aesculetin, beta-1,2-Mannotriose, Isocitric acid, Prunasin, Trehalose 6-phosphate, Eupatilin, 6β-Hydroxytestosterone, Stachyose, 2-Isopropylmalic acid, Gentisic acid, 5-Hydroxytryptophan, 5-Dihydroxybenzoic acid, 1H-Indole-3-acetamide, Galactitol, L-Theanine, Nitrobenzene, and 12-Hydroxydodecanoic acid.

Notably, for metabolites including Avermectin B1b aglycone, Sertraline, and Nitrobenzene (Fig. S8-S10)—whose presence in *P. sibiricum* seeds may appear unexpected and has not been previously reported—we provide justification supported by experimental rigor and metabolic logic: their identification was validated through strict metabolomic quality control (consistent retention times across six biological replicates and matching with multiple databases including HMDB and KEGG; Table S6), ensuring the reliability of detection, and their differential accumulation patterns during seed germination represent novel findings that warrant further investigation to fully understand their biological significance.

Among all identified differential metabolites, dAMP, Methyl jasmonate, Isocitric acid, and Galactitol showed continuous linear changes throughout germination (Fig. S11, Table S6), suggesting their critical roles in regulating seed germination. 

### Metabolic changes of carbohydrates and lipids

**Fig. 10:**
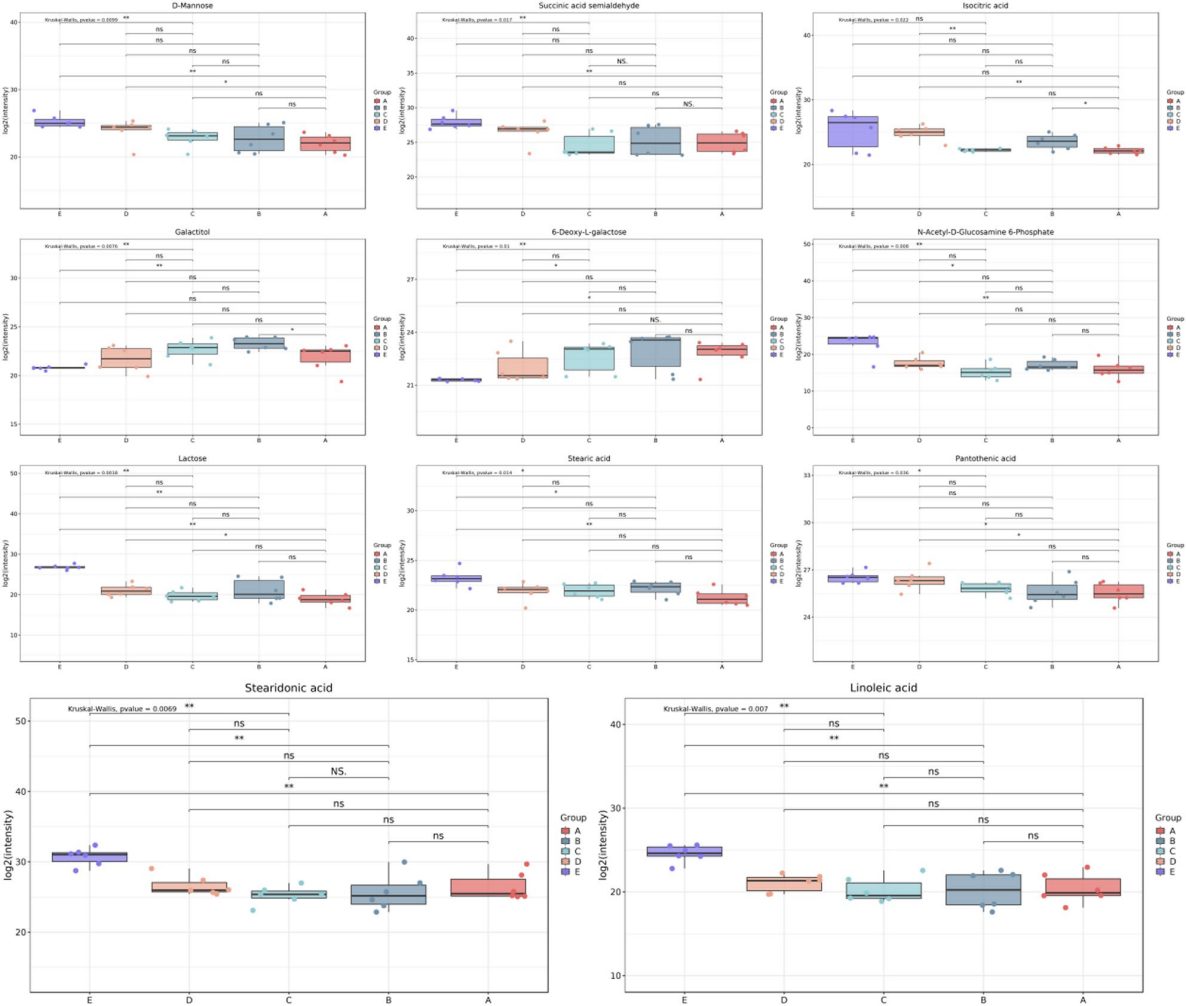
Differential metabolites of sugars and lipids during *P. sibiricum* seed germination(A: 0 d, B: 30 d, C: 60 d, D: 90 d, E: 105 d)

Carbohydrates and lipids, as core energy and material sources for *P. sibiricum* seed germination, exhibited distinct metabolic trends in this study (Fig. 10). Sugar metabolism provided essential energy and carbon skeletons: several sugar-related differential metabolites were identified, including cell wall components like D-Mannose, glycolysis intermediates such as succinic acid semialdehyde and Isocitric acid, and other sugars like 6-deoxy-L-galactose. Most of these sugar metabolites showed a downward trend (except 6-deoxy-L-galactose and Galactitol), which was linked to physiological processes of germination—D-Mannose degradation accompanied cell wall loosening and endosperm weakening, while Isocitric acid and similar metabolites were catabolized to supply energy for embryo elongation. Meanwhile, lipids (as key storage molecules) also participated in energy supply: four fatty acid synthesis-related differential metabolites (stearic acid, pantothenic acid, stearidonic acid, linoleic acid) showed a continuous downward trend throughout germination, reflecting the gradual degradation of stored lipids to release energy for supporting metabolic activities and growth during seed germination.

### KEGG enrichment analysis of differential metabolites

Investigating the enrichment pathways of differential metabolites is essential for understanding the changes in metabolic pathways during *P. sibiricum* seed germination [16]. These pathways encompass energy metabolism, material transport, signal transduction, cell cycle regulation, and other biological processes. Diverse biological activities and metabolic processes within organisms rely on the coordinated regulation of multiple genes. Enrichment analysis of KEGG pathways for all differential metabolites enables the identification of regulatory changes in the metabolic processes of *P. sibiricum* seeds during different germination stages. This study utilized KEGG database information to explore the biological pathways involved in germination. 

**Fig. 11:**
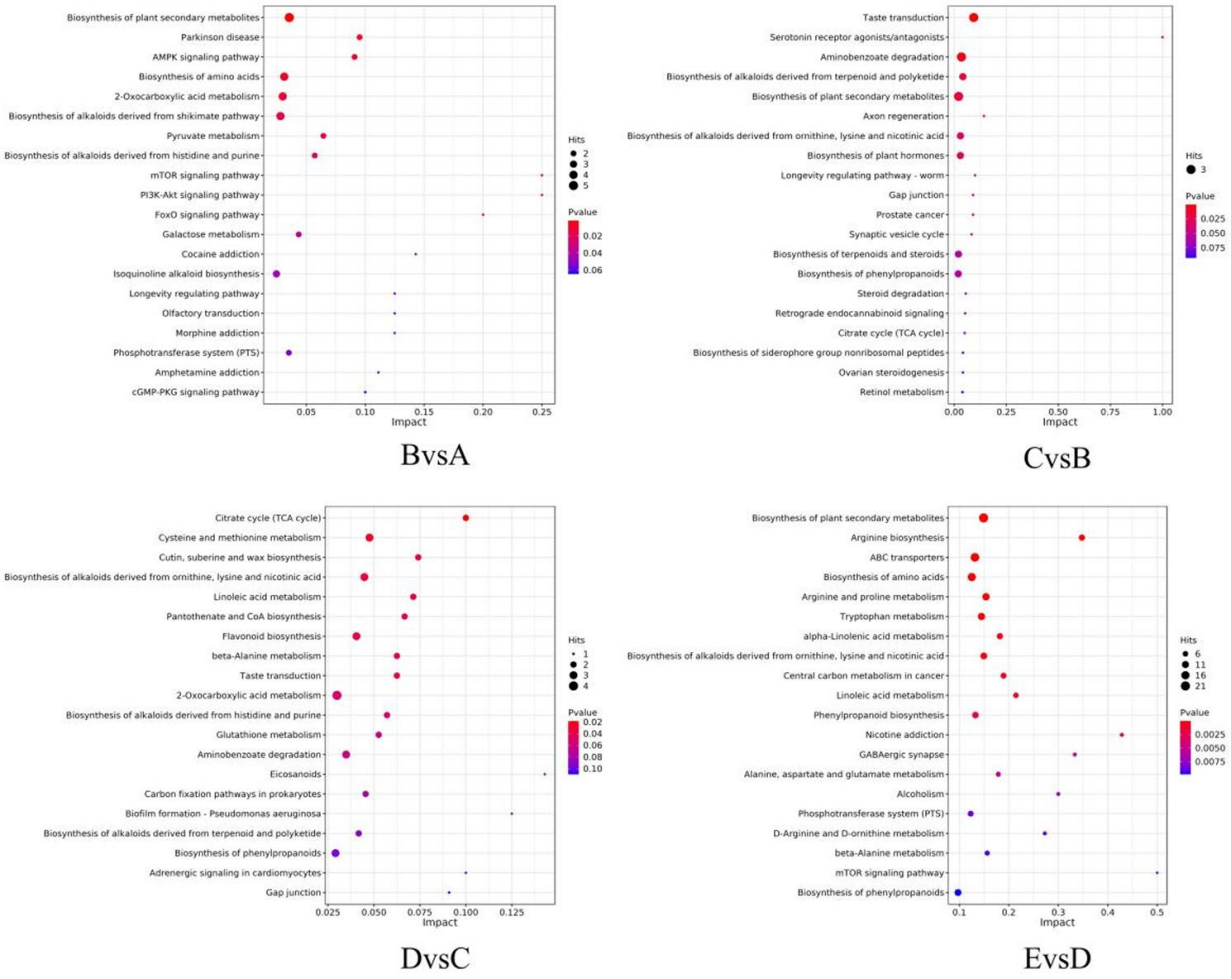
KEGG enrichment pathway(A: 0 d, B: 30 d, C: 60 d, D: 90 d, E: 105 d)

As shown in Fig. 11, the size of each circle represents the number of enriched differential metabolites, with larger circles indicating greater numbers. Circle colors indicate the reliability of metabolite enrichment within each pathway, with red signifying high reliability. The x-axis represents the enrichment impact value across different metabolic pathways, while the y-axis displays the names of the pathways. 

In BvsA, the top three enriched pathways were: Biosynthesis of plant secondary metabolites (map01060), Biosynthesis of alkaloids derived from the shikimate pathway (map01063), and 2-Oxocarboxylic acid metabolism (map01210). In CvsB, the top three pathways encompassed: Taste transduction (map04742), Aminobenzoate degradation (map00627), and Biosynthesis of plant secondary metabolites (map01060). In DvsC, the top three pathways were: 2-Oxocarboxylic acid metabolism (map01210), Cysteine and methionine metabolism (map00270), and Biosynthesis of alkaloids derived from ornithine, lysine, and nicotinic acid (map01064). In EvsD, the top three pathways included: Biosynthesis of plant secondary metabolites (map01060), ABC transporters (map02010), and Biosynthesis of amino acids (map01230). 

Among these pathways, the Biosynthesis of plant secondary metabolites was active throughout the germination process, while pathways like amino acid and protein biosynthesis contributed adequate energy for seed germination. 

### Transcriptome and metabolome association analysis 

A total of 68 key genes directly involved in seed germination were identified, and their associations with differential metabolites were analyzed using Mantel correlation analysis. For the gene dataset, we used their FPKM values across five germination stages (A-E) to calculate a dissimilarity matrix based on Euclidean distance. For the metabolite dataset, we used their normalized abundance values to compute a dissimilarity matrix also based on Euclidean distance. The Mantel test assessed the statistical significance of the correlation between the two matrices through 999 permutations, where a correlation coefficient (r) closer to 1 indicates a stronger correlation between the two datasets and a p-value closer to 0 indicates greater statistical significance of this correlation; thus, we set the screening criteria as p < 0.05 and r > 0.7 . 

The analysis revealed that 29 genes were significantly correlated with 41 metabolites that were involved in ascorbate and acetate metabolism, fructose and mannose metabolism, galactose metabolism, pathways and other processes related to cell wall synthesis and endosperm weakening. Furthermore, pathways associated with energy metabolism were identified, including fatty acid biosynthesis and degradation, glycerophospholipid, glycerol, starch, and sucrose metabolism, the pentose phosphate pathway, alkaloid biosynthesis via the shikimic acid pathway, and the citric acid cycle (TCA cycle). 

Differential metabolites were also linked to steroid phytohormone biosynthesis and signal transduction, phytohormone biosynthesis, and other regulatory pathways.

**Fig. 12:**
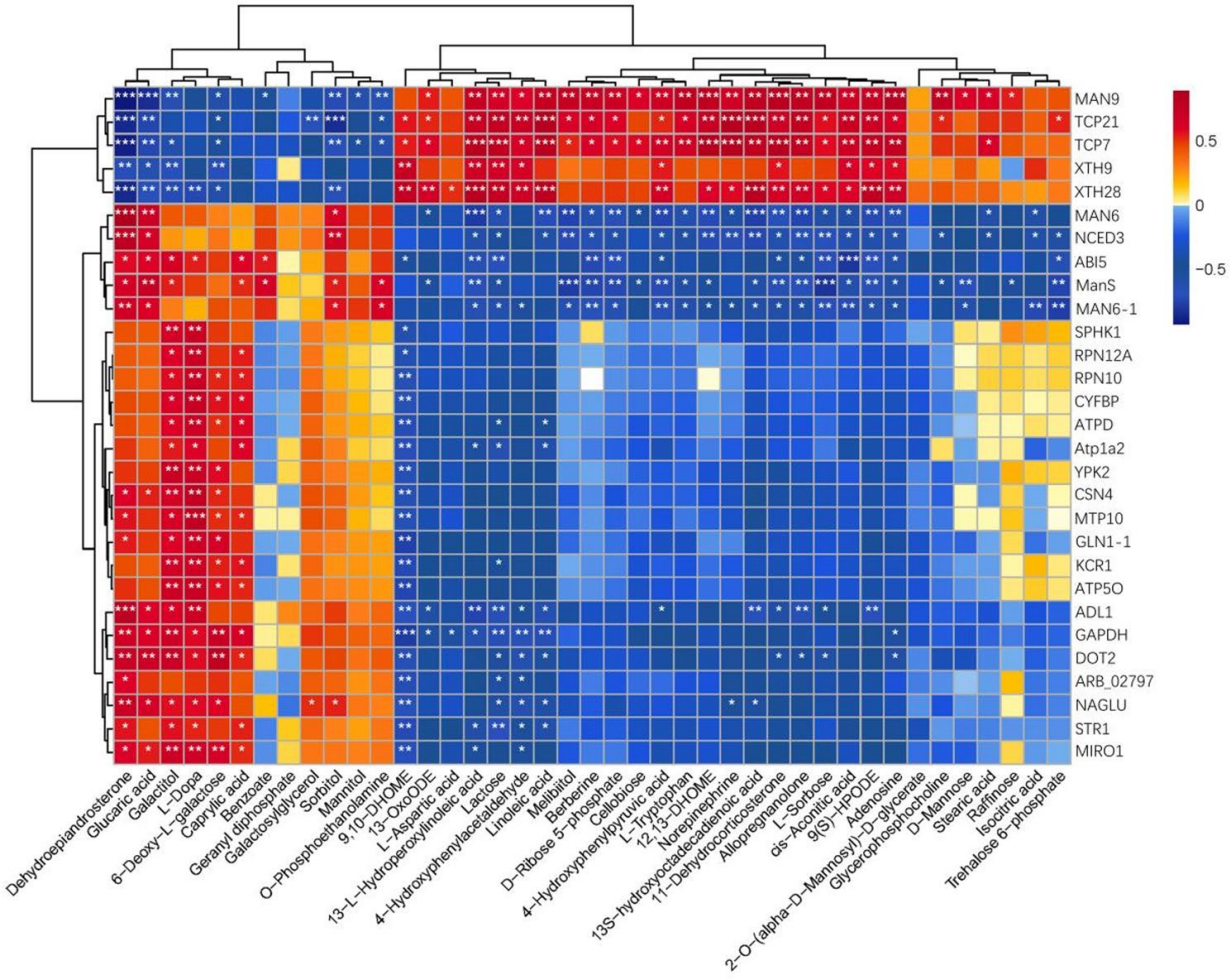
Heatmap of Mantel correlation analysis between key genes and differential metabolites

As shown in Fig. 12, *MAN9*, *TCP21*, *TCP7*, and *XTH28* were identified as the most influential genes among the 29 analyzed, which regulated the largest number of metabolites, with their regulatory patterns strongly consistent with known biological pathways. Specifically, *MAN9* (β-mannanase), a key enzyme in cell wall degradation, positively regulated lipid metabolites such as linoleic acid and 13-L-hydroperoxylinoleic acid while negatively regulating mannose-related polysaccharide metabolites, aligning with the classical pathway of cell wall loosening/endosperm weakening by degrading mannan in the cell wall to release monosaccharides and lipid precursors for embryo growth and phytohormone synthesis (e.g., jasmonic acid). *TCP21* and *TCP7*, members of the *TCP* transcription factor family localized in the nucleus, synergistically regulated dehydroepiandrosterone, sterol metabolites, and phytohormone-related metabolites such as methyl jasmonate, consistent with the plant transcription factor-mediated seed germination pathway where *TCP7*/*TCP21* binds to the promoters of downstream phytohormone synthesis genes (e.g., *ACX1*, *NCED3*) to regulate jasmonic acid and abscisic acid metabolism, thereby mediating the transition from seed dormancy to germination. *XTH28* (xyloglucan endotransglucosylase/hydrolase), a core gene in cell wall remodeling, positively regulated citric acid cycle (TCA cycle)-related metabolites (e.g., isocitric acid) and adenine metabolites (dAMP, AMP), aligning with the carbohydrate metabolism and energy supply pathway by loosening the cell wall to promote endosperm weakening while regulating carbohydrate degradation and the TCA cycle to improve ATP synthesis efficiency for seed germination. Among the 41 metabolites, Dehydroepiandrosterone, 13-L-Hydroperoxylinoleic acid, and Linoleic acid were the most significantly regulated. These findings suggested that *MAN9*, *TCP21*, *TCP7*, and *XTH28* played pivotal roles in the germination of *P. sibiricum* seeds, as did the essential activities of Dehydroepiandrosterone, 13-L-Hydroperoxylinoleic acid, and Linoleic acid. 

## Discussion 

Phytohormones, including ABA [17], IAA [18], and GA [19], regulate seed germination and emergence. GA plays a key role in seed germination across various species[20]. Cheng et al. reported that GA_3_ combined with low-temperature treatment (120 d at 0°C) may enhance the germination of *P. sibiricum* seeds [21]. Consistent with this, a recent hormonomics study on *P. sibiricum* further confirmed that 4°C cold stratification for over 70 d significantly alleviates double dormancy (embryo and physiological dormancy), and exogenous GA_3_ can synergize with cold stratification to promote germination by reconstructing the balance of endogenous phytohormones [22]. Notably, this study identified salicylic acid (SA) and ethylene as additional key regulatory phytohormones involved in the dormancy-germination transition, complementing the regulatory network of ABA/GA/IAA/JA/CTK identified in our study [22]. An analysis of the phytohormone content in our study revealed that ABA levels were elevated during dormancy, whereas GA_3_ and JA levels peaked at the seed coat breakthrough stage, while IAA exhibited an increasing trend—this hormone dynamic is consistent with the changing pattern observed in the closely related species *Polygonatum cyrtonema*, where ABA, active GAs, IAA, and JA gradually decrease with the progression of germination to match the physiological demand for endosperm weakening [23]. Transcriptomic changes were associated with phytohormone signal transduction as well as starch metabolism and other carbohydrate-related pathways [24], which is supported by proteomic and transcriptomic data from *P. cyrtonema* showing that these pathways are consistently enriched during key germination stages (imbibition, radicle emergence, cotyledon elongation) [23].

Numerous genes are involved in seed germination, from encoding regulatory factors and sensor complexes to mediating downstream metabolic reactions, all of which are influenced by phytohormones, carbohydrates, and environmental factors [25, 26]. RNA-seq analysis identified numerous DEGs in seeds across various germination stages, with a total of 1,710 genes involved in the germination process and implicated in biological pathways including cell wall loosening, endosperm weakening, primary metabolism, phytohormone biosynthesis, signal transduction, radicle growth, and early seedling development. A recent proteomic study on *P. cyrtonema* highlighted that endosperm weakening is a critical limiting step for germination, which is precisely consistent with our scanning electron microscopy observations of gradual endosperm degradation [23]. Similar to rapeseed, where cell wall modification, carbohydrate metabolism, and phytohormone-related pathways were enriched with DEGs during germination [27], *P. sibiricum* also shares this conserved genetic basis, reflecting the universal gene regulatory networks underlying seed germination. Additionally, fluridone-mediated dormancy breaking in *P. cyrtonema* further confirmed that phytohormone signal transduction and energy metabolism are core regulatory pathways for germination, and our study also identified phenylpropanoid biosynthesis (a key branch of secondary metabolism) as a highly enriched pathway, which is consistent with the findings of this study [28].

LC-MS analysis results were consistent with gene expression data, and this multiomics integration identified beta-1,2-mannotriose, isocitric acid, dAMP, AMP, and methyl jasmonate as key metabolic markers for *P. sibiricum* seed germination. Differential metabolic pathway analysis revealed that seed germination was regulated by multiple interconnected metabolic pathways, and metabolic correlation analysis further confirmed the interdependence between germination-related metabolites and these pathways [29]. Starch can serve as a source of energy for germination when it is hydrolyzed into glucose [30], and as a critical carbon source, sugar supports seed germination by providing both energy and biosynthetic precursors [31]. It has been reported that the accumulation of degraded starch and soluble sugars increases during the transition from seed germination to emergence, which is crucial for stimulating seed vigor expression [32]. During seed germination, starch degradation is an important biochemical mechanism that can increase the water retention and osmotic pressure within cells to provide energy for seed germination [33]. Moreover, fluridone treatment in *P. cyrtonema* significantly upregulated genes related to the tricarboxylic acid (TCA) cycle, further verifying that enhanced energy metabolism efficiency is essential for supporting seed germination [28]. Amino acid metabolism is essential as it must coordinate with carbohydrate metabolism to promote cellular processes and facilitate seed germination [34]. Consistent with this, enrichment analysis revealed that plant secondary metabolism, amino acid synthesis, and carbohydrate metabolism were highly enriched at each germination stage. 

## Conclusions 

This study systematically investigated the dormancy release and germination mechanisms of *P. sibiricum* seeds using integrated morphological, phytohormonal, transcriptomic, and metabolomic approaches. Germination conditions were optimized using response surface methodology, yielding a maximum germination rate of 89.31% ± 0.34% under the optimized parameters (GA_3_ 130.497 mg/L, 4.90°C, 1:1 sand–Huangjiang slag ratio), providing a practical basis for large scale seed propagation.

Multi omics integration identified 68 key genes involved in cell wall loosening, endosperm weakening, energy metabolism, hormone signaling, and transcriptional regulation. Their differential expression drove dynamic shifts in downstream metabolites, including sugars, lipids, and hormone related compounds, thereby constructing a gene–metabolite regulatory network that governs the transition from dormancy to germination. Among these, four core genes—*MAN9*, *TCP21*, *TCP7*, and *XTH28*—were identified as pivotal regulators linked to distinct metabolite sets: *MAN9* was associated with linoleic acid, 13 hydroperoxylinoleic acid, and mannose related polysaccharides; *TCP21* and *TCP7* were correlated with dehydroepiandrosterone and methyl jasmonate; and *XTH28* was associated with TCA cycle intermediates and adenine derivatives.

These regulators formed two functionally coordinated modules: the *MAN9*–*XTH28* module, which coordinates cell wall remodeling with carbohydrate and energy metabolism, and the *TCP21*–*TCP7* module, which mediates hormone metabolism and the germination transition. Beyond its conserved role in cell wall degradation, *MAN9* exhibited a novel regulatory function in lipid metabolism by modulating jasmonic acid precursors, representing a functional diversification not previously reported in medicinal plants. These findings establish the first gene–metabolite regulatory framework for *P. sibiricum* seed germination, advancing mechanistic understanding from physiological traits to a systematic molecular level.

Comparative analyses further highlight the novelty of the identified gene–metabolite pairs (e.g., *MAN9*–linoleic acid, *TCP21*–methyl jasmonate), which have not been documented in previous studies. Collectively, these results demonstrate that seed germination in *P. sibiricum* is a complex process governed by crosstalk between environmental cues and endogenous programs. This work provides a foundational molecular framework for future studies aimed at improving germination efficiency and molecular breeding in this medicinal species.

## Supplementary Information

Supplementary Material 1.

## Data Availability

The raw sequence data (RNA-seq) generated in this study are available in the NCBI Sequence Read Archive (SRA) under BioProject accession number PRJNA1391072 (https://www.ncbi.nlm.nih.gov/bioproject/PRJNA1391072).The metabolomics data have been deposited to the MetaboLights database with the identifier MTBLS14022 (https://www.ebi.ac.uk/metabolights/MTBLS14022).All other data are contained within the article and Supplementary files.

## Abbreviations

ANOVA: Analysis of variance
CTK: Cytokinin
CV: Coefficient of Variation
DEGs: Differentially expressed genes
Em: Embryo
En: Endosperm
Ge: Germ
Sc: Seed coat
ABA: Abscisic acid
GA_3_: Gibberellic acid 3
IAA: Indole-3-acetic acid
JA: Jasmonic acid
KEGG: Kyoto Encyclopedia of Genes and Genomes
KOG: euKaryotic Orthologous Groups
LC-MS: Liquid Chromatography-Mass Spectrometry
MAN: β-mannanase
NR: Non-redundant protein
qRT-PCR: Real Time quantitative PCR
RNA-seq: RNA sequencing
TTC: Triphenyltetrazolium chloride
