# Supplementary material for: Integrative transcriptomic and metabolomic analysis reveals key genes and regulatory networks underlying seed germination in Polygonatum sibiricum
Source: BMC Genomics. 2026 Apr 15;27:503. doi: 10.1186/s12864-026-12803-x (PMC13191861; doi:10.1186/s12864-026-12803-x)
Supplement: Supplementary file 1 — Supplementary Material 1. [file 12864_2026_12803_MOESM1_ESM.docx]

Table S1 Reaction system of qPCR

| Reagent | Volume (μL) |
| --- | --- |
| SYBR Premix Ex Taq™ II | 10 |
| BsbZIP forward primer (10 μM) | 0.5 |
| BsbZIP reverse primer (10 μM) | 0.5 |
| RNase-Free H_2_O | 4 |
| cDNA ( < 100 ng) | 5 |

Table S1 Note: This table presents the composition of the 20 μL reaction system used for qRT-PCR validation of genes related to seed germination in *Polygonatum sibiricum* Red. The internal reference gene was *TUB*, and all reagents were standard molecular biology reagents. The cDNA template concentration was controlled within 100 ng. Each experiment was performed with three technical replicates, and the mean values were used for subsequent analysis.

Table S2 Reaction procedure of qPCR

| Step | Condition |
| --- | --- |
| Pre-denaturation | 95°C, 1 min |
| Denaturation | 94°C, 30 s |
| Annealing | 58°C, 30 s |
| Extension | 72°C, 1 min |
| Cycle number | 35 cycles |
| Final extension | 72°C, 2 min |

Table S2 Note: This table shows the reaction program for qRT-PCR of genes related to seed germination in *Polygonatum sibiricum* Red. A three-step amplification protocol was performed for 35 cycles on a real-time PCR system. Melt curve analysis was used to verify amplification specificity. The experiment was conducted with three technical replicates.

Table S3 qRT-PCR primers

| Primer Name | Sequence |
| --- | --- |
| *NCED3-*F | GGCCATCGAAGGGCTGTACC |
| *NCED3-*R | GCCGGAGATCTGGACGTTCG |
| *CYC11-*F | ATGTCGAAGGCGCGTGTGAA |
| *CYC11-*R | GGCTCAACAGTAGCCGGTCAA |
| *VHA-E1-*F | GAAGCGCAGGAGAAGGCCAA |
| *VHA-E1-*R | GCGAGCAATGCGCTTGTTGA |
| *ATP5O-*F | CCGCCAAGTTCTCCAAGCCA |
| *ATP5O-*R | AGGTCACCTTCGCCTGGACT |
| *atpA-*F | GTGGGTTACCAGCCAACGCT |
| *atpA-*R | TTGCAGCACCAGCATCACCA |
| *atp5b-*F | GTGGGTTACCAGCCAACGCT |
| *atp5b-*R | TCGCCTGCACCGACGTAATC |
| *ABI5-*F | GAGGTCCCGCGCTAGGAAAC |
| *ABI5-*R | CCTCCTTGCCTGCGTCCTTT |
| *4CLL7-*F | AGAAGGGCGATGCTGTCGTG |
| *4CLL7-*R | CTTGGCGGACGTCACTTGGT |
| *TCP7-*F | CCCGCGTCTTCCAACTGACC |
| *TCP7-*R | GACTGGGTGCTGGAGCTGAC |
| *TCP21-*F | GAAGGACCGGCACTCGAAGG |
| *TCP21-*R | AGGAGCCACTCGATGGTCTGT |

Table S3 Note: This table lists the specific primer sequences used for qRT-PCR validation of genes related to seed germination in *Polygonatum sibiricum* Red. The internal reference gene was *TUB*.

Table S4 ANOVA of response surface model

| **Source** | **Sum of Squares** | **df ^a^** | **Mean Square** | **F-value** | **p-value** |
| --- | --- | --- | --- | --- | --- |
| Model | 2133.11 | 11 | 193.92 | 2058.88 | < 0.0001 |
| A-Phytohormone concentration | 9.46 | 1 | 9.46 | 100.45 | 0.0002 |
| B-Stand storage temperature | 24.26 | 1 | 24.26 | 257.53 | < 0.0001 |
| C-sand storage substrate | 508 | 2 | 254 | 2696.75 | < 0.0001 |
| AB | 39.88 | 1 | 39.88 | 423.41 | < 0.0001 |
| AC | 27.63 | 2 | 13.81 | 146.67 | < 0.0001 |
| BC | 29.62 | 2 | 14.81 | 157.25 | < 0.0001 |
| A^2^ | 604.75 | 1 | 604.75 | 6420.78 | < 0.0001 |
| B^2^ | 690.07 | 1 | 690.07 | 7326.62 | < 0.0001 |
| Residual | 0.4709 | 5 | 0.0942 |  |  |
| Lack of Fit | 0.177 | 1 | 0.177 | 2.41 | 0.1956 |
| Pure Error | 0.2939 | 4 | 0.0735 |  |  |
| Cor Total | 2133.58 | 16 |  |  |  |
| R^2^ = 0.9998  0.988544  0.988544 | *Adj*R^2^ = 0.9993 |  |  | C.V.% =0.4204 |  |

Table S4 Note: This table presents the analysis of variance (ANOVA) results for the response surface model (BBD design) optimizing germination conditions for *Polygonatum sibiricum* seeds. Key statistical parameters, including R^2^, adjusted R^2^, and coefficient of variation (C.V.%), were used to evaluate model fit. A p-value < 0.05 was considered statistically significant, and p < 0.0001 was considered highly significant.

Table S5 Summary of *Polygonatum sibiricum* Sequencing Data Quality

| sample | raw_reads | raw_bases | clean_reads | clean_bases | Q20 (%) | Q30 (%) |
| --- | --- | --- | --- | --- | --- | --- |
| A1 | 49333908 | 7449420108 | 43419842 | 6556396142 | 96.8 | 92.59 |
| A2 | 43994942 | 6643236242 | 39392944 | 5948334544 | 96.92 | 92.86 |
| A3 | 42129444 | 6361546044 | 37963450 | 5732480950 | 96.95 | 92.82 |
| B1 | 42953244 | 6485939844 | 37963450 | 5942756000 | 96.96 | 92.93 |
| B2 | 44807164 | 6765881764 | 40487126 | 6113556026 | 96.93 | 92.83 |
| B3 | 42860224 | 6471893824 | 39357932 | 5943047732 | 97.11 | 93.14 |
| C1 | 42107720 | 6358265720 | 38630046 | 5833136946 | 97.21 | 93.21 |
| C2 | 44052724 | 6651961324 | 39923134 | 6028393234 | 97.12 | 93.16 |
| C3 | 40939720 | 6181897720 | 37336746 | 5637848646 | 96.98 | 92.77 |
| D1 | 45839296 | 6921733696 | 40695192 | 6144973992 | 96.87 | 92.72 |
| D2 | 40390020 | 6098893020 | 36375528 | 5492704728 | 96.94 | 92.76 |
| D3 | 44556954 | 6728100054 | 39852878 | 6017784578 | 97 | 92.85 |
| E1 | 47351410 | 7150062910 | 42629084 | 6436991684 | 96.89 | 92.77 |
| E2 | 42135924 | 6357408400 | 38114484 | 5750602830 | 97.14 | 93.11 |
| E3 | 44765388 | 6759573588 | 39871086 | 6020533986 | 97.16 | 93.01 |

Table S4 Note: This table presents the statistics of raw data and clean data obtained from transcriptome sequencing of *Polygonatum sibiricum* seeds at five germination stages (A-E). Three biological replicates were set for each stage. The sequencing data quality was high, as indicated by Q20 ≥ 96% and Q30 ≥ 92%, ensuring suitability for subsequent de novo assembly and differential gene expression analysis.

Table S6 26 key differential metabolites during *Polygonatum sibiricum* seed germination

| **Name** | **ID** | **FC** | **p-value** | **posneg** |
| --- | --- | --- | --- | --- |
| Homocitric acid | M207T181 | 2.43 | 0.025974 | pos |
| Methyl jasmonate | M207T757 | 3.4 | 0.041126 | pos |
| 4-(2-Aminophenyl)-2,4-dioxobutanoic acid | M208T470 | 2.19 | 0.041126 | pos |
| Ferreirin | M303T650 | 15.6 | 0.041126 | pos |
| Sertraline | M306T282 | 2.34 | 0.008658 | pos |
| (S)-Reticuline | M329T449 | 4.48 | 0.041126 | pos |
| dAMP | M332T147 | 7.13 | 0.041126 | pos |
| Berberine | M336T607 | 7.81 | 0.041126 | pos |
| AMP | M348T228 | 2.15 | 0.041126 | pos |
| (13E)-11a-Hydroxy-9,15-dioxoprost-13-enoic acid | M353T696 | 13.64 | 0.004329 | pos |
| Avermectin B1b aglycone | M570T583 | 10.91 | 0.002165 | pos |
| Nitrobenzene | M122T332 | 2.06 | 0.008658 | neg |
| Gentisic acid | M153T195 | 2.34 | 0.041126 | neg |
| Galactitol | M163T233 | 2.21 | 0.041126 | neg |
| L-Theanine | M173T148 | 2.15 | 0.041126 | neg |
| 1H-Indole-3-acetamide | M174T533 | 2.33 | 0.025974 | neg |
| 2-Isopropylmalic acid | M175T88 | 2.35 | 0.041126 | neg |
| Aesculetin | M177T768 | 341.45 | 0.002165 | neg |
| Isocitric acid | M192T67 | 3.22 | 0.041126 | neg |
| 12-Hydroxydodecanoic acid | M215T714 | 2.01 | 0.008658 | neg |
| Prunasin | M295T142 | 3.11 | 0.041126 | neg |
| 6beta-Hydroxytestosterone | M303T805 | 2.75 | 0.015152 | neg |
| Eupatilin | M343T731 | 2.83 | 0.041126 | neg |
| Trehalose 6-phosphate | M421T74_1 | 2.86 | 0.025974 | neg |
| beta-1,2-Mannotriose | M485T102 | 8.79 | 0.015152 | neg |
| Stachyose | M665T102_2 | 2.43 | 0.025974 | neg |

Table S4 Note: This table lists the 26 core differential metabolites identified during the seed germination process of *Polygonatum sibiricum* seed. The screening criteria were p < 0.05 and |FC| > 2. The table includes detection results from both positive and negative ion modes, with IDs representing the characteristic metabolite numbers in the LC-MS analysis (pos = positive ion mode, neg = negative ion mode).


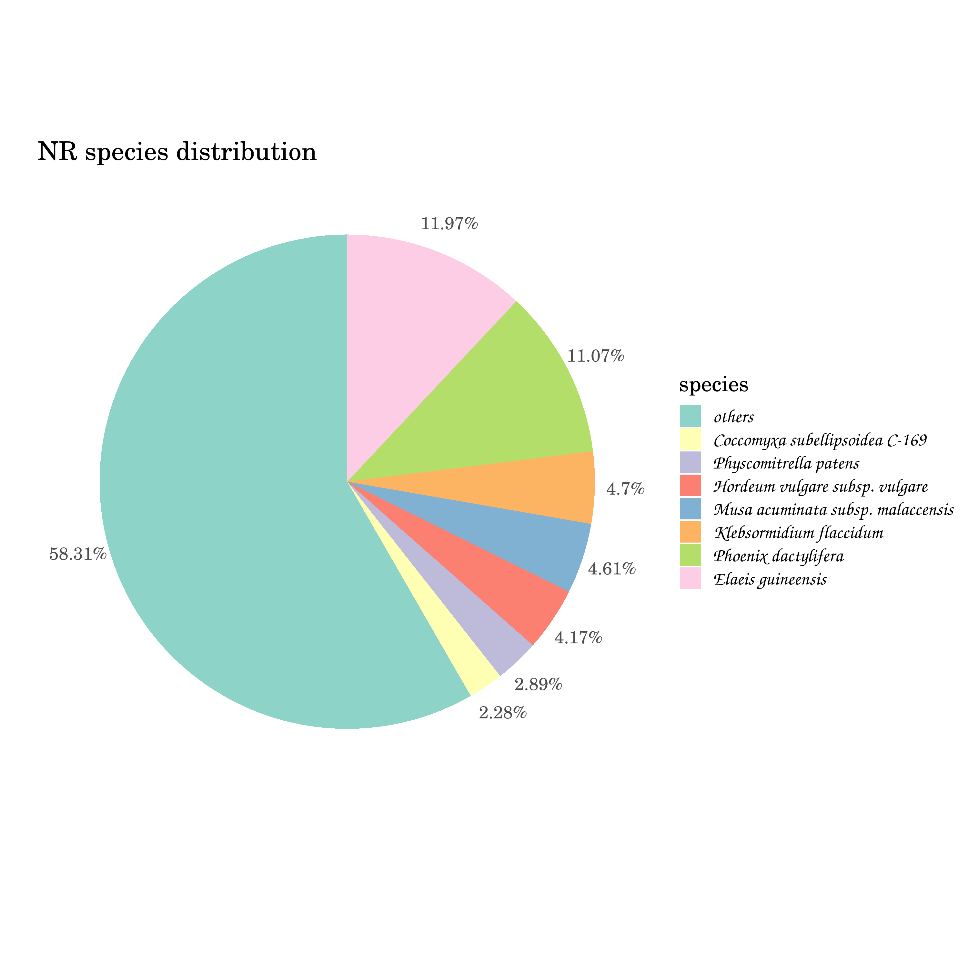


Figure S1 Species distribution of unigenes annotated in NR database

Figure S1 Legend: This figure shows the species homology distribution of *Polygonatum sibiricum* seed germination transcriptome unigenes based on BLASTX alignment against the NR database. The horizontal axis represents plant species with high homology, and the vertical axis represents the percentage of unigenes matching each species. A total of 58.31% of the unigenes were annotated to other plant species, indicating relatively low homology between *Polygonatum sibiricum* and currently sequenced species, thus necessitating de novo transcriptome assembly.


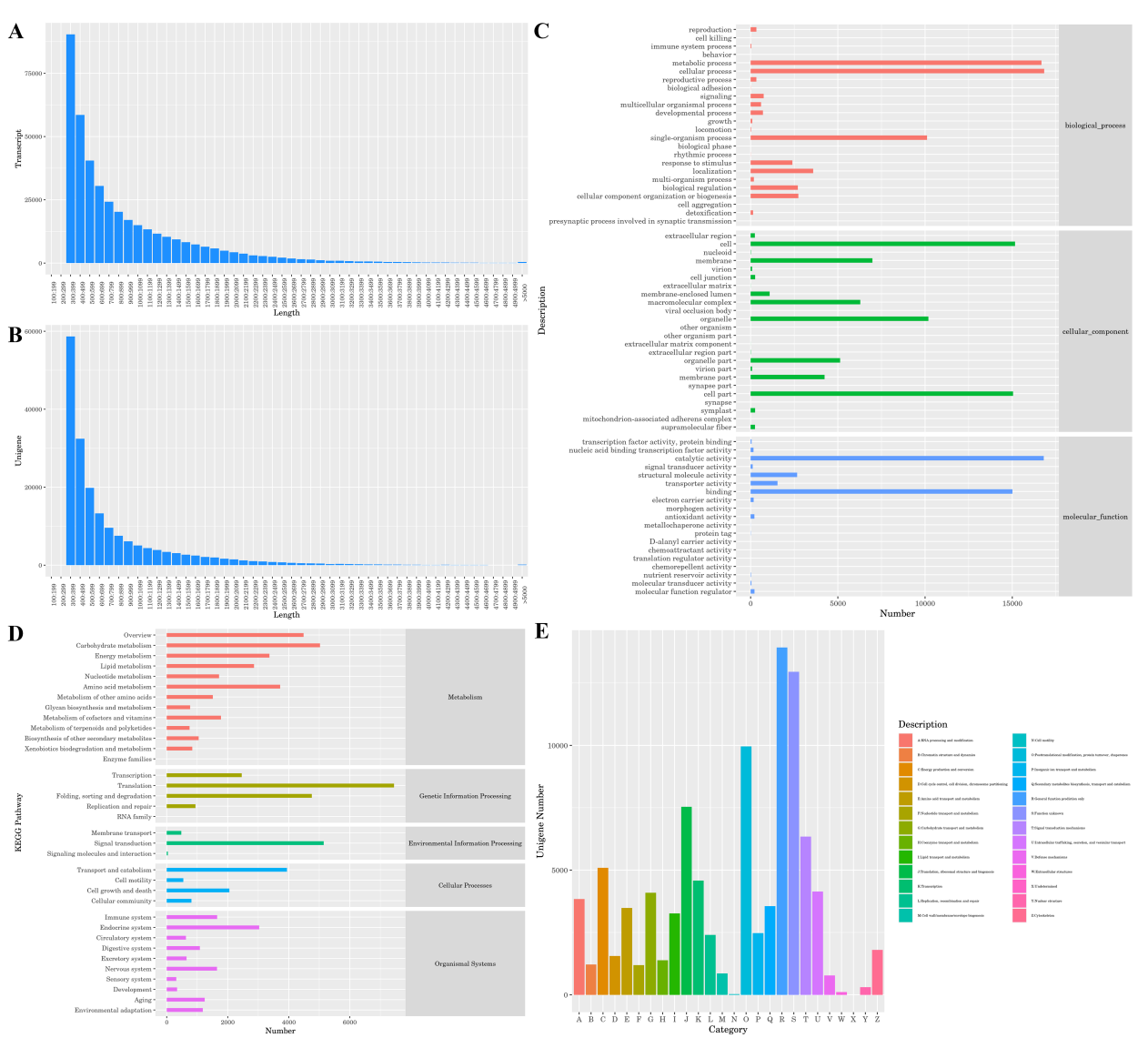


Figure S2 Transcript and unigene annotation statistics of *Polygonatum sibiricum* seed transcriptome

Figure S2 Legend: Statistics of assembly and annotation results for the *Polygonatum sibiricum seed* germination transcriptome. (A) Length distribution of transcripts; (B) Length distribution of unigenes; (C) Annotation statistics from the GO database (categorized into three main groups: biological process, cellular component, and molecular function); (D) Annotation statistics from the KEGG database (classified by metabolic pathways); (E) Annotation statistics from the eggNOG database (classified by functional clusters). The vertical axis represents the number of sequences, and the horizontal axis represents the classification/length interval.


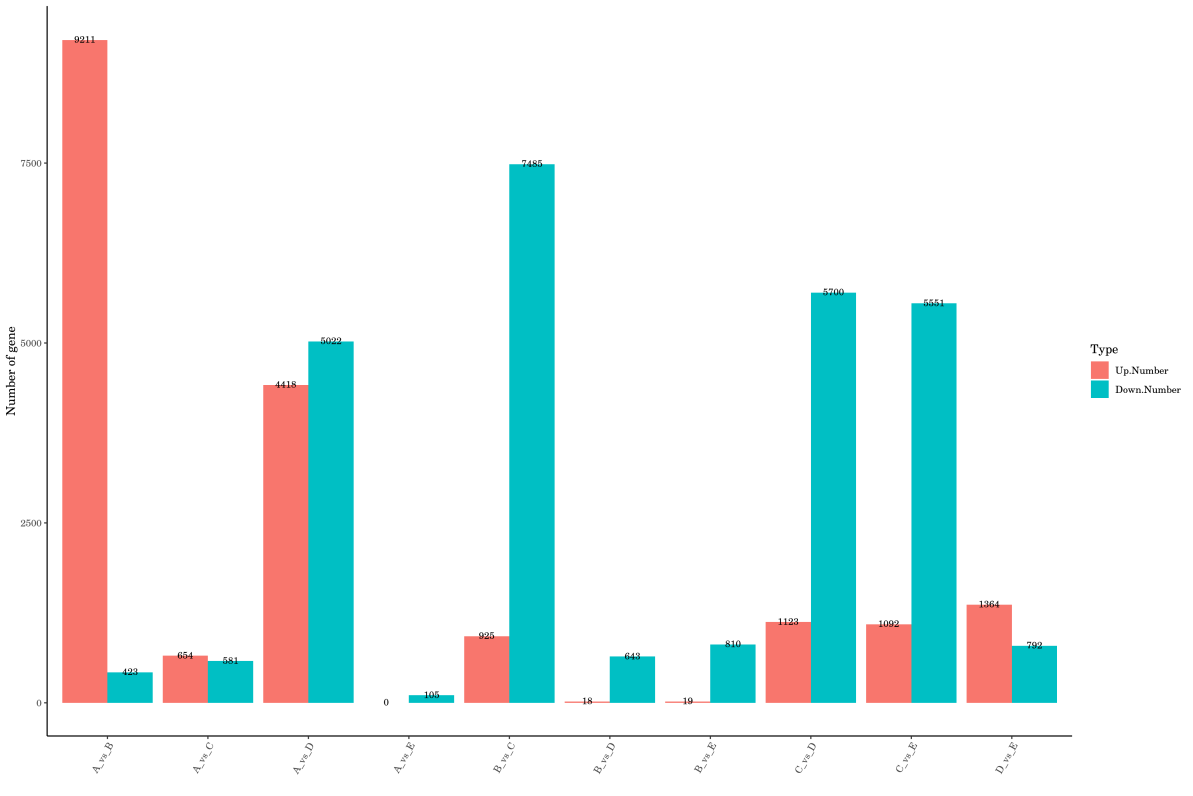
Figure S3 Statistics of differentially expressed genes (DEGs) in pairwise comparison of *P. sibiricum* seed germination stages

Figure S3 Legend: Statistics of DEGs from pairwise comparisons among the five germination stages of *Polygonatum sibiricum* seeds. (A) Bar graph showing the number of differentially expressed genes (Up.Number = number of up-regulated genes, Down.Number = number of down-regulated genes); (B) Pairwise comparison matrix. The horizontal axis represents the comparison groups, and the vertical axis represents the number of genes. DEGs were identified using the screening criteria of FDR < 0.05 and |log_2_FC| > 1.


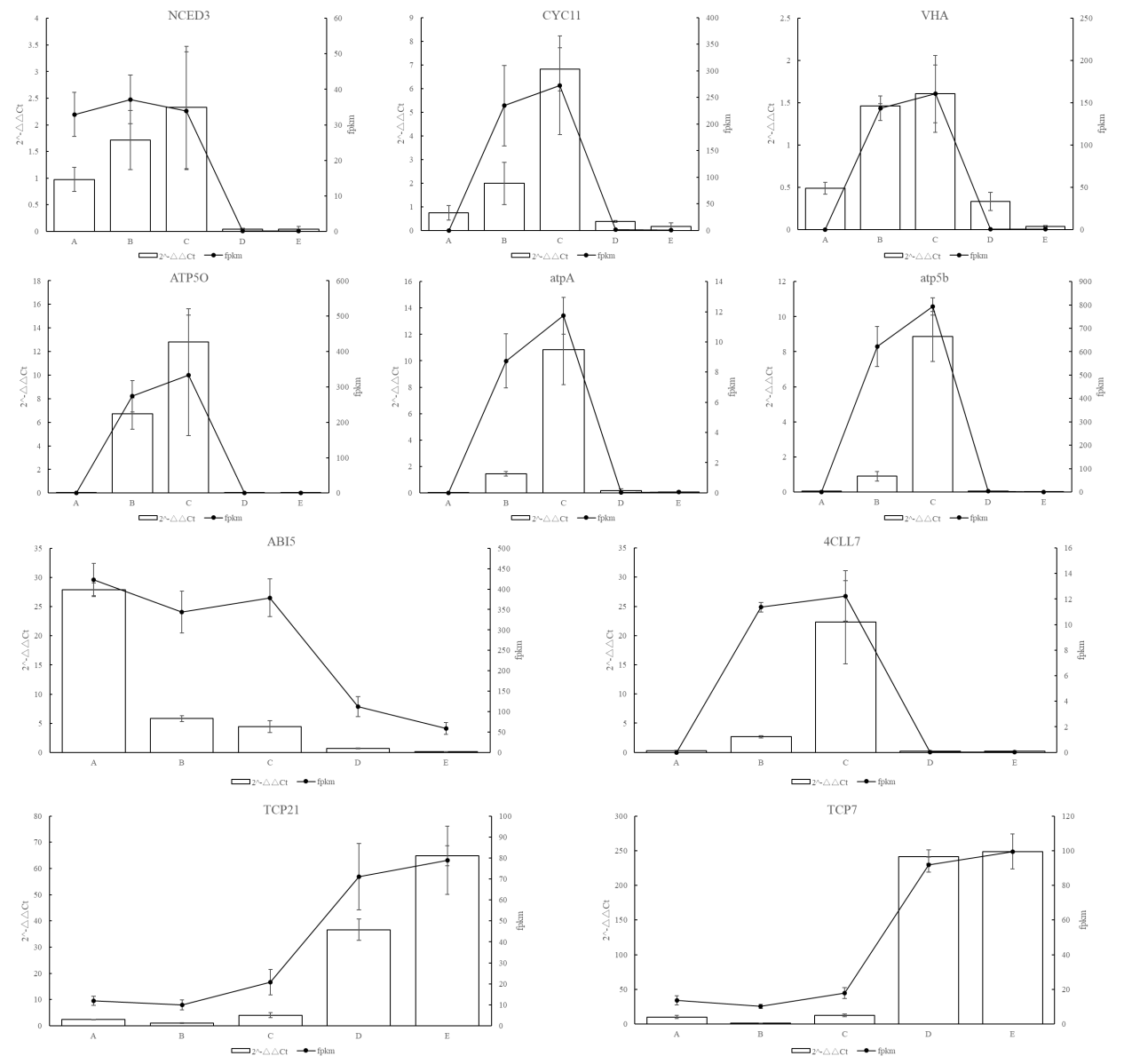


Figure S4 qRT-PCR validation of 10 key DEGs during *Polygonatum sibiricum* seed germination

Figure S4 Legend: Validation of 10 key differentially expressed genes related to *Polygonatum sibiricum* seed germination by qRT-PCR. The horizontal axis represents the germination stages (A: 0 d, B: 30 d, C: 60 d, D: 90 d, E: 105 d).


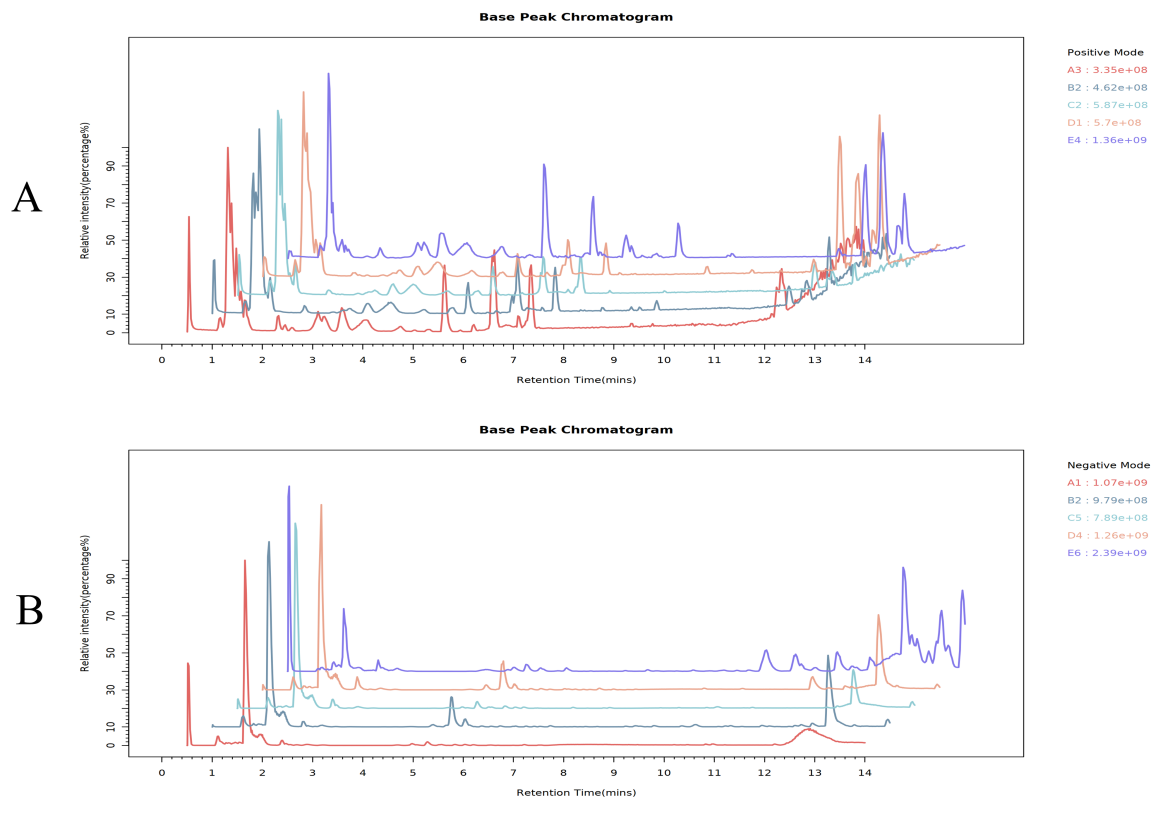


Figure S5 Total ion chromatogram (TIC) of *Polygonatum sibiricum* seed metabolites detected by LC-MS

Figure S5 Legend: Total ion current (TIC) chromatograms of *Polygonatum sibiricum* seed germination metabolome detected by LC-MS. (A) Positive ion mode; (B) Negative ion mode. The horizontal axis represents retention time (min), and the vertical axis represents ion intensity.


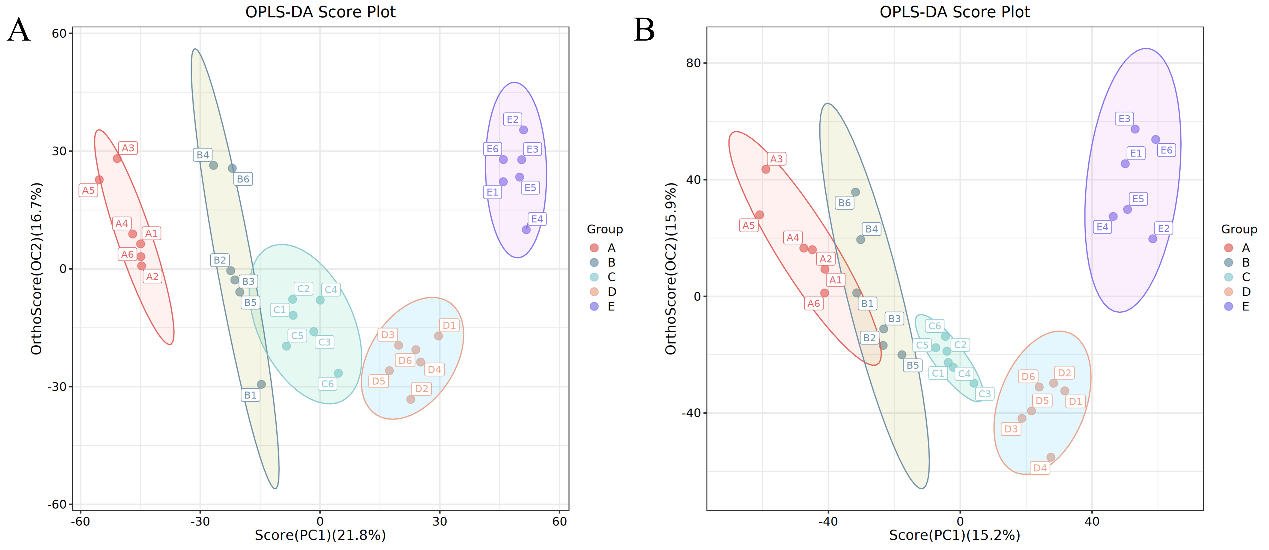


Figure S6 OPLS-DA score plot of metabolite profiles in *Polygonatum sibiricum* seed germination stages

Figure S6 Legend: Orthogonal partial least squares discriminant analysis (OPLS-DA) score plots of metabolite profiles at different stages of *Polygonatum sibiricum* seed germination. (A) Positive ion mode; (B) Negative ion mode. Different colors/symbols represent the five germination stages (A: 0 d, B: 30 d, C: 60 d, D: 90 d, E: 105 d).


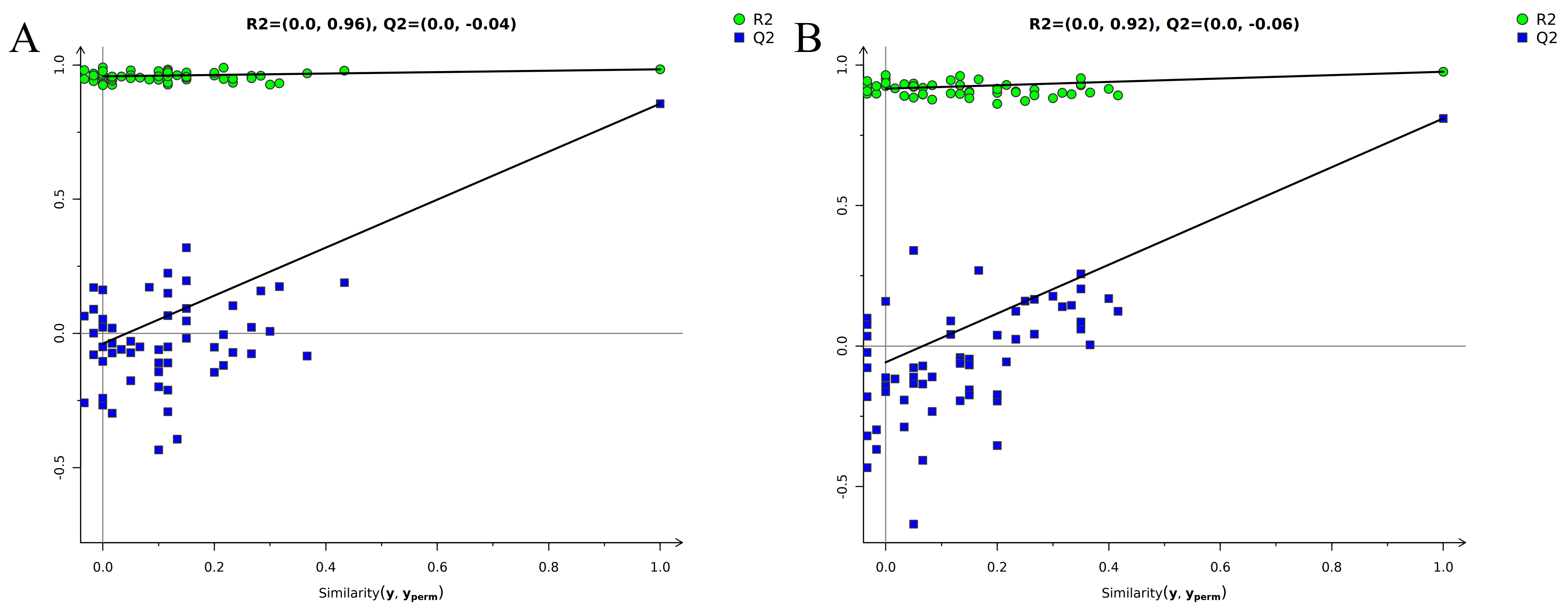
Figure S7 Permutation test of OPLS-DA model for *Polygonatum sibiricum* seed metabolome

Figure S7 Legend: Permutation test plots of the OPLS-DA model for the *Polygonatum sibiricum* seed metabolome, used to validate whether the model is overfitted. (A) Positive ion mode; (B) Negative ion mode. The horizontal axis represents similarity (y, y_perm), and the vertical axis represents R^2^ (model interpretability) and Q^2^ (model predictability).


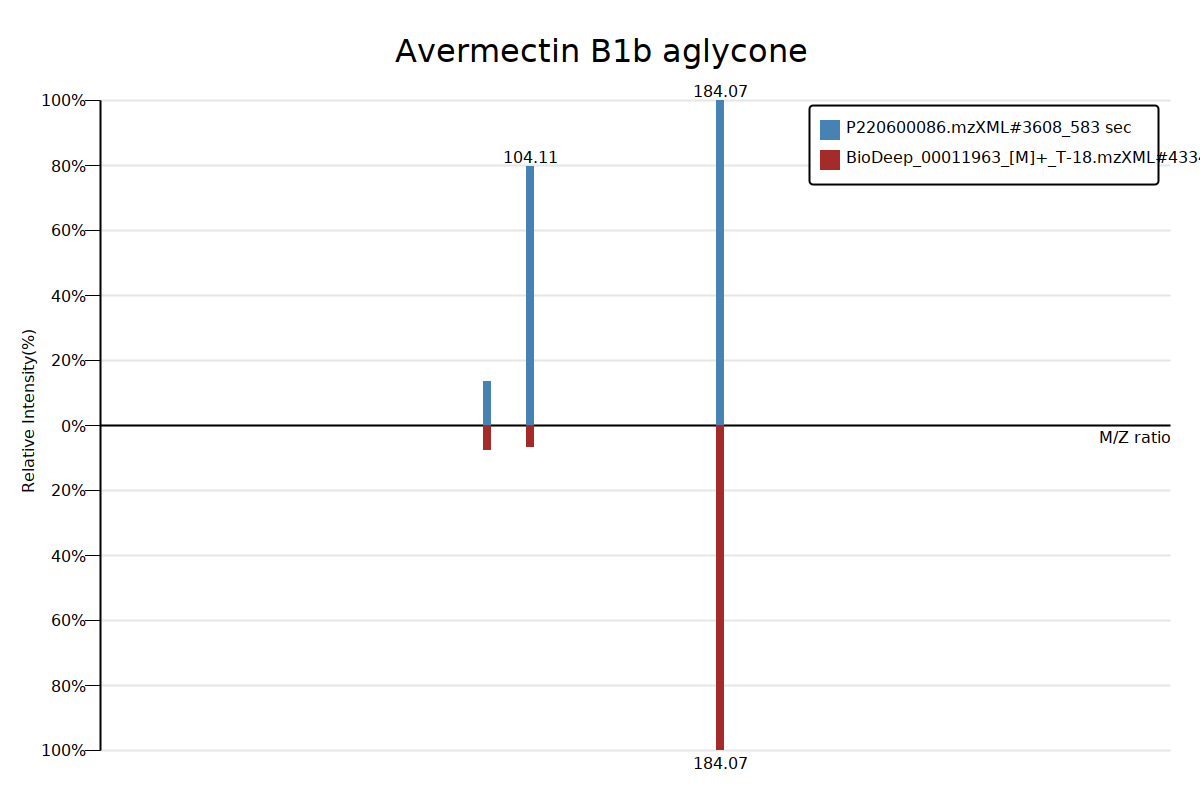


Figure S8 MS/MS spectra of Avermectin B1b aglycone


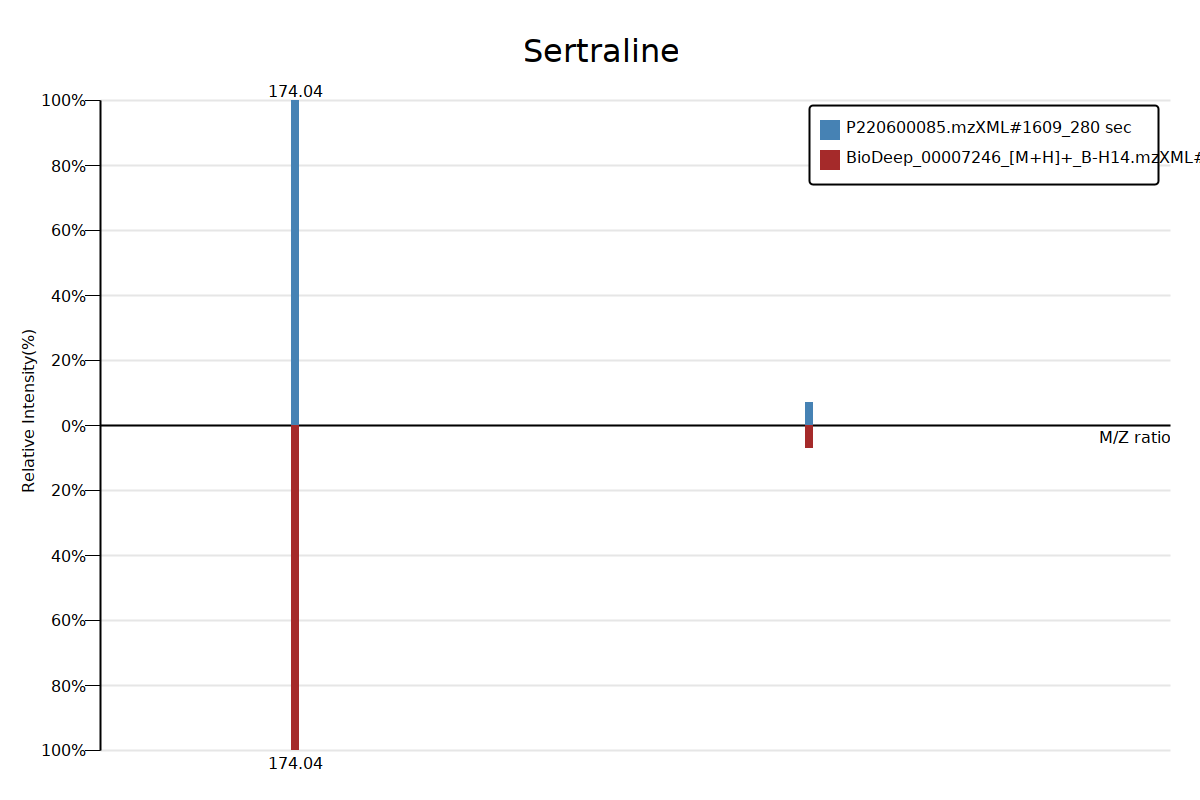


Figure S9 MS/MS spectra of Sertraline


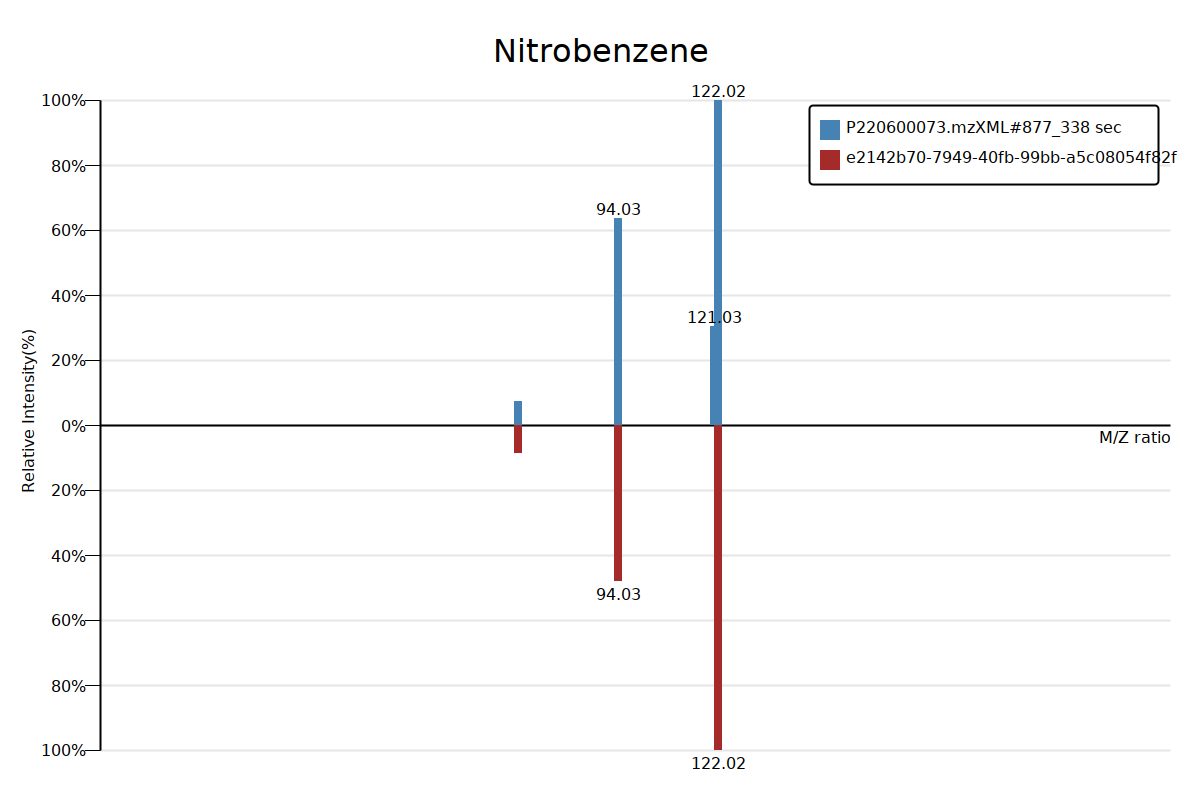


Figure S10 MS/MS spectra of Nitrobenzene


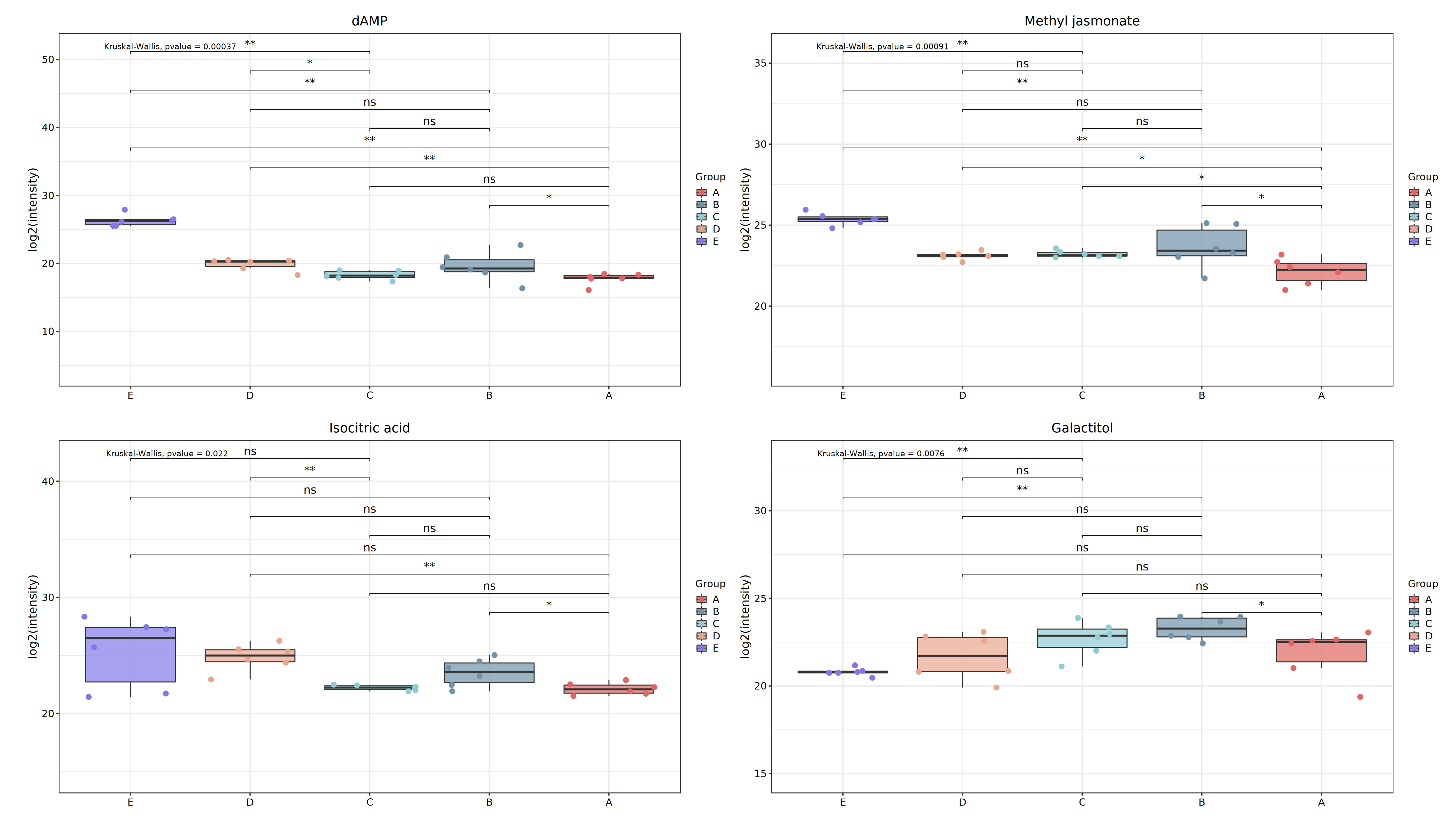


Figure S11 Box plot of four key differential metabolites during *Polygonatum sibiricum* seed germination

Figure S11 Legend: Box plots of four core differential metabolites during *Polygonatum sibiricum* seed germination. The horizontal axis represents the germination stages (A: 0 d, B: 30 d, C: 60 d, D: 90 d, E: 105 d), and the vertical axis represents the relative metabolite content.
